# Can EMG-Derived Upper Limb Muscle Synergies Serve as Markers for Post-Stroke Motor Assessment and Prediction of Rehabilitation Outcome?

**DOI:** 10.3390/s25103170

**Published:** 2025-05-17

**Authors:** Fung Ting Kwok, Ruihuan Pan, Shanshan Ling, Cong Dong, Jodie J. Xie, Hongxia Chen, Vincent C. K. Cheung

**Affiliations:** 1 School of Biomedical Sciences, and Gerald Choa Neuroscience Institute, The Chinese University of Hong Kong, Hong Kong, China; 1155125931@link.cuhk.edu.hk (F.T.K.); jodiexie825@gmail.com (J.J.X.); 2 The Second Affiliated Hospital, Guangzhou University of Chinese Medicine, and The Guangdong Provincial Hospital of Chinese Medicine, Guangzhou 510000, China; panruihuan@126.com (R.P.); ling33@gzucm.edu.cn (S.L.); amydong0516@outlook.com (C.D.); 3 Joint Laboratory of Bioresources and Molecular Research of Common Diseases, The Chinese University of Hong Kong and Kunming Institute of Zoology of the Chinese Academy of Sciences, Hong Kong, China

**Keywords:** factorization, motor control, motor modules, motor primitives, patient stratification, precision rehabilitation

## Abstract

EMG-derived muscle synergy, as a representation of neuromotor modules utilized for motor control, has been proposed as a biomarker for stroke rehabilitation. Here, we evaluate the utility of muscle synergies for assessing motor function and predicting post-intervention motor outcome in a stroke rehabilitation clinical trial. Subacute stroke survivors (n = 59) received month-long acupuncture (Acu), sham acupuncture (ShamAcu) or no acupuncture (NoAcu) as adjunctive rehabilitative intervention alongside standard physiotherapy. Clinical scores and EMGs (14 muscles, eight motor tasks) were collected from the stroke-affected upper limb before and after intervention. We then extracted muscle synergies from EMGs using non-negative matrix factorization and designed 12 muscle synergy indices (MSIs) to summarize different aspects of post-stroke synergy features. All MSIs correlated with multiple clinical scores, suggesting that our indices could potentially serve as biomarkers for post-stroke motor functional assessments. While the intervention groups did not differ in their pre-to-post differences in the clinical scores, the inclusion of MSIs into analysis revealed that on average Acu promoted more recovery of synergy features than ShamAcu and NoAcu, though not all subjects in the group were Acu responders. We then built regression models using pre-intervention MSIs and clinical variables to predict the outcomes of Acu and NoAcu and showed by a preliminary retrospective simulation of patient stratification that MSI-based predictions could have led to better post-intervention motor improvement. Overall, we demonstrate that muscle synergies can potentially clarify the effects of interventions and assist in motor assessment, outcome prediction, and treatment selection.

## 1. Introduction

Stroke is a prevalent neurological disorder and a leading cause of adult disability. It occurs when blood flow to the brain is disrupted by a blockage (ischemic stroke) or rupture (hemorrhagic stroke) [1]. Since motor dysfunction is a common complication of stroke, motor rehabilitation is crucial for restoring quality of life post-stroke. However, the development of new rehabilitation strategies beyond conventional approaches for stroke survivors has been challenging. Despite the positive results of early trials and meta-analyses, recent large-sample multicenter clinical trials testing novel rehabilitation therapies showed neutral results in the primary endpoints [2]. Consequently, there is a need to improve the efficacy of rehabilitation therapies, potentially through the utilization of neurological biomarkers to elucidate the underlying treatment mechanisms, assign stroke survivors with heterogeneous pre-rehabilitation presentations to suitable treatment modalities, and customize treatment duration and content for individual stroke survivors [3,4,5]. Ideally, biomarkers for motor rehabilitation should align well with the structure of the motor system and the neurological principles of motor control [6,7].

**Figure 1:**
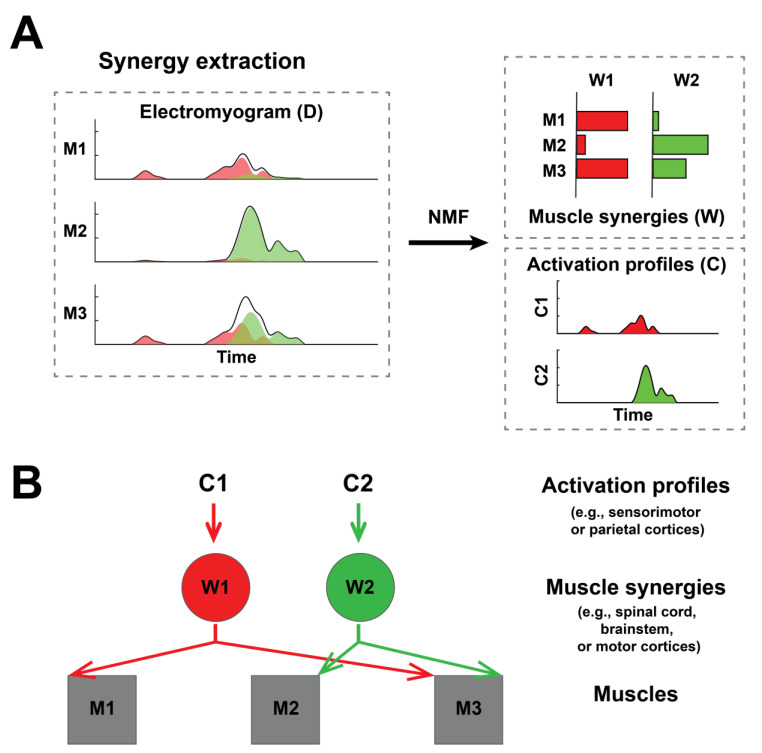
The concept of muscle synergies. This simplified example shows 2 synergies (synergy 1 in red and synergy 2 in green) for 3 muscles (M1–M3). (**A**) A schematic of synergy extraction. In our study, the preprocessed EMG of every session was decomposed into muscle synergies (i.e., the W matrix) and activation profiles (i.e., the C matrix) using non-negative matrix factorization (NMF). (**B**) A schematic for muscle synergies and their activation profiles. To illustrate, we have drawn three muscles (M1–M3), and two muscle synergies (W1–W2) and their corresponding activation profiles (C1–C2). Muscle synergies represent low-level neuronal networks such as those in the spinal cord or brainstem, while their activation profiles represent neural commands encoded by higher motor areas such as the sensorimotor cortex.

Muscle synergies can be one such biomarker. In contrast to EEG or MRI, where the activities or structure of the brain are directly measured, muscle synergy is an electromyographic (EMG) data-derived representation of the neuromotor modules of the motor system, thereby shedding light on the underlying motor strategies an individual uses to orchestrate multi-muscle coordination during different movement tasks. To identify muscle synergies, multi-muscle EMG data (the **D** matrix) is decomposed into muscle synergies (the **W** matrix) and their activation profiles (the **C** matrix) using factorization algorithms such as the non-negative matrix factorization (NNMF) [8] (Figure 1A). According to the muscle synergy model, the motor system is able to generate diverse movements at an affordable computational load because it activates groups of muscles organized in synergies described above, as opposed to individual muscles [9,10].

Physiologically, muscle synergies extracted from EMG during voluntary movements reflect the connectivity between neurons within the spinal cord, brainstem, and/or motor cortices and the motoneuronal pools of the muscles (Figure 1B). This is supported by studies demonstrating its resemblance to the muscle fields of spinal premotor neurons in monkeys [11], or to the muscle synergies extracted from EMG elicited from stimulation of the motor cortices in monkeys [12] or humans [13]. On the other hand, the activation profiles of muscle synergies may represent neural drives encoded by higher motor areas such as the sensorimotor or parietal cortices [10] (Figure 1B).

In the past decade, muscle synergy analysis has been progressively refined to study the pathophysiological basis of movement disorders such as stroke [14] (Supplementary Table S1). Previous studies that explore the use of muscle synergies as a stroke rehabilitation biomarker have developed indices to quantify multiple pathologically relevant synergy features [15,16,17,18,19,20,21,22,23,24,25,26,27,28,29,30], which can be classified into six categories (Supplementary Table S1). The results of these studies were promising but also limited in that in many studies, only a subset of synergy features were shown to correlate with clinical scores, likely due to the relatively small sample size (with an average of 15 stroke survivors [14]) or limited number of synergy features included. Indeed, many studies focused more on the characteristics of spatial synergies (categories 1–3 in Supplementary Table S1) than temporal coefficients (categories 4–5) [14].

**Figure 2:**
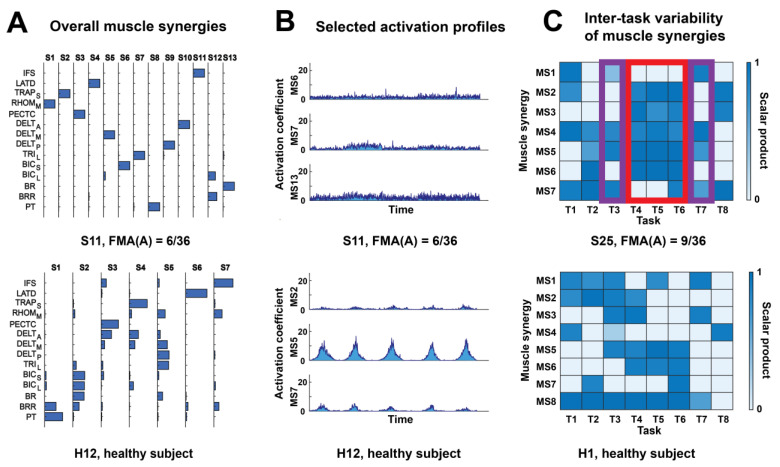
Post-stroke alterations in muscle synergies and activation profiles. Subjects with the most distinguishable synergy features were chosen to illustrate our point here. (**A**) The overall muscle synergies of a severely impaired stroke survivor (S11, FMA(A) = 6/36) and a healthy subject (H12). S11 had a greater number of muscle synergies, and the synergy compositions were more fragmented. (**B**) The selected activation profiles (i.e., those of the three most activated synergies in task 8) of the same pair of subjects. The activation profile of subject S11 exhibited more oscillations, more irregularity, and a smaller area under curve. (**C**) The inter-task variability of muscle synergies of another severely impaired stroke survivor (S25, FMA(A) = 9/36) and healthy subject (H1) pair. Each task-specific muscle synergy was matched with an overall muscle synergy of the same subject on a one-to-one basis. The colormaps represent the scalar products between each task-specific synergy and the matched overall synergy. We used red and purple boxes to highlight the tasks in which S25 used almost the same synergy combinations (red box: synergies 2 + 3 + 4 + 5 + 6 ± 7 were recruited in tasks 4–6; purple boxes: synergies 1 + 4 + 5 + 7 were recruited in both task 3 and task 7).

In this study, we aimed to evaluate the clinical utility of muscle synergies as a biomarker for the assessment and prediction of stroke rehabilitation on a clinical trial of acupuncture delivered as an adjunctive rehabilitative intervention for subacute stroke survivors. First, we identified the synergy features that potentially correlated with post-stroke motor impairments (Figure 2). We then designed indices to quantify those features and validated them by correlating the indices with multiple clinical scores using the pre-intervention data of the stroke survivors participating in the clinical trial (n = 59) together with data from additional stroke survivors (n = 29) for a larger sample size.

We then used the MSIs designed to reveal how motor functional improvements were achieved after acupuncture. Acupuncture is an ancient technique widely adopted in China as an adjunct therapy to promote motor recovery after stroke. Although it has been shown to benefit survivors of ischemic stroke in animals [31] and humans [32,33], the underlying mechanism is not yet fully understood. Here, we are not only interested in the clinical effect of acupuncture, but also whether the motor recovery it induces may be attributed to the recovery of normal synergy patterns or the emergence of compensatory synergy patterns.

Lastly, we tested the ability of the pre-intervention MSIs to predict the rehabilitation outcome of acupuncture. Neurological biomarkers, such as those used in the Predict REcovery Potential (PREP) algorithm, can predict the extent to which stroke survivors respond to conventional upper limb rehabilitation [34]. Another study demonstrated that the efficacy of acupuncture is contingent upon the lesion size in the periventricular white matter [35]. Additionally, stroke survivors who responded to rehabilitation exhibited distinct pre-intervention muscle synergy features in comparison to those who did not [36]. We therefore hypothesized that MSIs could predict the motor functional outcome of individual stroke survivors after acupuncture, and that MSI-based predictive models could be used to stratify patients into subject groups who should or should not receive acupuncture alongside basic physiotherapy for the best rehabilitation outcome.

## 2. Methods

### 2.1. Subject Recruitment

The study was approved by the Ethics Committee of the Guangdong Provincial Hospital of Chinese Medicine (initial: NO. BF-2018-164-01, approval date: 30 November 2018; revised: NO. BF-2018-164-02, approval date: 19 January 2020). All participants gave informed consent prior to experimentation. Between January 2019 and May 2022, we recruited 59 first-onset, hemiparetic stroke survivors at their subacute stage of recovery (age = 57.14 ± 11.14 years; female = 23; post-stroke duration = 47.59 ± 26.88 days) to participate in a randomized clinical trial of acupuncture (no. NCT03712085 at ClinicalTrials.gov) in which they were randomly assigned to receive acupuncture (Acu) (n = 21), sham acupuncture (ShamAcu) (n = 21), or no acupuncture (NoAcu) (n = 17) alongside basic care for one month (Supplementary Tables S2 and S3). The inclusion and exclusion criteria for RCT subjects are listed in Supplementary Note S1.

The Acu and ShamAcu groups received, alongside basic care, the therapeutic and sham versions of Bo’s abdominal acupuncture [37,38], respectively, which lasted for 30 min per day, 5 days per week, for 1 month. The NoAcu group received basic care only. Basic care consisted of conventional upper limb rehabilitative training and measures for secondary stroke prevention. For the Acu group, abdominal needles (diameter = 0.2 mm and length = 30 mm; Suzhou Hualun Medical Appliance Co., Ltd., Suzhou, China) were inserted into eight abdominal acupuncture points, including Guanyuan (RN4), Qihai (RN6), Xiawan (RN10), Zhongwan (RN12), ipsilateral Shangqu (KI17), ipsilateral Huaroumen (ST24), ipsilateral Shangfengshidian (AB1), and ipsilateral Shangfengshiwaidian (AB2). For the ShamAcu group, customized flat needles (diameter = 0.3 mm and length = 30 mm; Changzhou Dayi Medical Device Co., Ltd., Changzhou, China) were inserted into the same acupoints to produce a placebo effect.

We further recorded data from an additional 29 stroke survivors with similar characteristics (age = 54.38 ± 10.48 years; female = 8; post-stroke duration = 70.10 ± 43.03 days) to increase the statistical power of our analysis of the pre-intervention data (Supplementary Tables S2 and S3). In addition, we also recorded EMG from 10 adults with no history of neurological disorders (age = 28.00 ± 4.92; female = 6) to serve as a reference for normal synergy characteristics and a template for MSI computation (see below). All stroke survivors and control subjects in this study were right-handed.

### 2.2. Clinical Assessments

To assess the motor performance of stroke survivors, we used multiple clinical assessments from both the body function/impairment and the activity/disability domains under the International Classification of Functioning, Disability and Health (ICF) [39], including the Fugl-Meyer Assessment of the Upper Extremity (FMA(UE)) and the Brunnstrom Stage (BS) for the former domain, and the Wolf Motor Function Test (WMFT) and the modified Barthel Index (BI) for the latter (Supplementary Table S4). We calculated two additional clinical scores, FMA(A) and BI(UE), by selecting a subset of task items from FMA(UE) (i.e., section A) and BI (i.e., feeding, dressing, grooming, bathing, toileting, and chair-bed transfers), respectively (Supplementary Table S4). This is because we extracted muscle synergies from the EMG activities of muscles responsible for shoulder, arm, or forearm movements (see below), and the selected items correspond to the functions of the recorded muscles.

For all stroke survivors in the RCT trial (n = 59), clinical assessments (i.e., FMA, BS, WMFT, and BI) were conducted repeatedly at weeks 0 (pre-intervention), 2 (half-completed intervention), and 4 (post-intervention). For the rest of the stroke survivors not in RCT (n = 29), FMA(UE) and BI were evaluated in a single session upon recruitment.

To reflect the impact of the interventions on the clinical scores of each RCT subject, we calculated both the absolute changes in clinical scores (i.e., post-treatment score minus pre-treatment score) and the realized recovery after intervention, which is defined as the actual change in clinical score relative to the maximum possible score improvement [40,41]:

$$Realized\;Recovery=\frac{{score}_{post}-{score}_{pre}}{{score}_{max}-{score}_{pre}}$$.

### 2.3. EMG Recording and Extraction of Muscle Synergies

We recorded surface EMGs from 14 muscles of the stroke-affected upper limbs of stroke survivors (at week 0 for all stroke survivors, and at week 4 for those who participated in the RCT), along with EMGs from each of both upper limbs of healthy subjects, which served as a normative baseline for comparison. Electrodes were placed following SENIAM guidelines for skin preparation, placement, fixation, and testing of sensor connection [42]. The recorded muscles were as follows: infraspinatus (IFS); latissimus dorsi (LATD); trapezius, superior fibers (TRAP_S_); rhomboid major (RHOM_M_); pectoralis major, clavicular head (PECTC); deltoid, anterior part (DELT_A_); deltoid, middle part (DELT_M_); deltoid, posterior part (DELT_P_); triceps, lateral head (TRI_L_); biceps, short head (BIC_S_); biceps, long head (BIC_L_); brachialis (BR); brachioradialis (BRR); and pronator teres (PT). As a result, we recorded 167 EMG sessions, including 88 pre-intervention sessions (for all stroke survivors) and 59 post-intervention sessions (for stroke survivors in the RCT), as well as 20 sessions from healthy subjects (10 sessions for each side). During EMG recording, the subjects performed 8 upper limb motor tasks related to activities of daily living (with each task repeated 5 times, thus resulting in 40 episodes): simple upward reaching, shoulder abduction, forward reaching across a single spatial constraint, upward reaching across two constraints, hand pronation, shoulder circumduction, moving along a path together with hand pronation, and shoulder extension [15,16]. All EMGs were preprocessed with filtering, rectification, integration, and variance normalization as described previously [43].

For every EMG session, we used nonnegative matrix factorization (NNMF) [44] to extract two types of muscle synergies, namely, a set of overall muscle synergies from the concatenated EMGs across all tasks (i.e., 8 tasks × 5 repetitions per task = 40 episodes) and 8 sets of task-specific muscle synergies from the EMG of each task (i.e., 5 episodes), to reflect the global and task-specific data structures, respectively. Specifically, NNMF decomposes an EMG matrix (**D**) into time-invariant muscle synergies (**W**) and their activation profiles (**C**) (Figure 1A), such that $D^{mxt}=W^{mxn}*C^{nxt}+error$, where *m* = number of muscles recorded (i.e., 14 in our study), *n* = predefined number of muscle synergies (i.e., 1, 2, …, up to 14), and *t* = number of time points (each representing 20 ms after preprocessing). For each synergy extraction, NNMF was applied to successively extract 1, 2, …, up to 14 muscle synergies because 14 muscles were simultaneously recorded. For each number of synergies, NNMF was repeated 50 times using initial **W** and **C** randomized between 0 and the maximum EMG value; each repetition was terminated when the change in R^2^ was < 0.001% for 20 consecutive iterations. The synergy set with the highest R^2^ was chosen to represent the corresponding number of synergies in downstream analysis. For the overall muscle synergies, the optimal dimensionality was defined as the minimum number of muscle synergies that attained an R^2^ > 0.80 in *both* the all-task EMG and each of the 8 single-task EMGs. For task-specific muscle synergies, the optimal dimensionality was defined as the minimum number of synergies that yielded an R^2^ > 0.80 for the single-task EMG.

### 2.4. Rationale and Computation of Muscle Synergy Indices

We designed 12 muscle synergy indices (MSIs; described below) to concisely quantify the post-stroke characteristics of the muscle synergies (**W**) and their activation profiles (**C**). For each stroke survivor, we calculated the 12 MSIs for every EMG session. Some of the MSIs (i.e., DO, MEA, ITV_BFRR_W_, ITV_BFRR_C_, and ITV_BFRR_C_ (mod)) were computed solely using the **W**s and **C**s of the stroke-affected upper limbs. Other MSIs (i.e., DevD_O_, DevD_A_, BFRR_W_, BFRR_C_, and BFRR_C_ (mod)) were computed by comparing the **W**s and **C**s of stroke survivors with those of healthy subjects, with the final MSI value being the average across healthy subjects. In order to account for the potential left–right differences in muscle synergies, the affected limb of each stroke survivor was compared with the side-matched healthy limbs. Indices MI and FI were computed by comparing the **W** of each stroke survivor with the centroids of healthy synergy clusters. For all the above MSIs, baseline values were calculated from the **W**s and **C**s of every EMG session of healthy subjects analogously.

The computations of the MSIs are described in detail in Supplementary Note S2 and summarized below and in Table 1. Briefly, indices DevD_O_ and DevD_A_ represent the post-stroke deviation of the number of muscle synergies from the normative; BFRR_W_ and BFRR_C_ or BFRR_C_ (mod) assess the normalcy of the **W** and **C** matrices after stroke, respectively; MI and FI quantify the extent of post-stroke muscle synergy merging and fractionation, respectively; DO and MEA calculate the degree of oscillation and the magnitude of activation in the activation profile, respectively; and ITV_BFRR_W_ and ITV_BFRR_C_ or ITV_BFRR_C_ (mod) determine the variability of **W** or **C** across tasks, respectively, with higher values indicating smaller inter-task variability. While we computed some MSIs (indices DevD_O_, DevD_A_; MI, FI) in a similar way as previous studies, we developed new methodologies to capture the post-stroke synergy characteristics reported by previous studies (indices BFRR_W_, BFRR_C_, and BFRR_C_ (mod); ITV_BFRR_W_, ITV_BFRR_C_, and ITV_BFRR_C_ (mod)) and quantify new features observed in our stroke survivors (indices DO, MEA).

*Indices DevD_O_*, *DevD_A_.*

Dimensionality refers to the number of muscle synergies. DevD_O_ and DevD_A_ were calculated by taking the original and absolute values of the difference in dimensionality between stroke survivors (D_S_) and healthy subjects (D_R_), respectively:

$${DevD}_{O}=D_{S}-D_{R};\;{DevD}_{A}=\left|D_{S}-D_{R}\right|$$.

*Index BFRR_W_*.

To evaluate the normalcy of the **W** matrix of a stroke survivor’s affected limb, previous studies used scalar products [15,16,17,18,19,20,21,22,23,24,25,26,27,28] or unidirectional fitting [17] to compare the stroke-affected **W** matrix with that of a reference limb (i.e., the unaffected limb of the same patient or the limb of a healthy subject). These approaches may yield misleading results when the dimensionalities (i.e., number of synergies) of the stroke-affected limb and the reference limb differ, because of the following reason. In prior studies that relied on a scalar product for evaluation, the muscle synergies of the affected limb were matched to the synergies of the reference limb based on maximum scalar product, and the scalar products across matched synergy pairs were often combined into an index for the normalcy of the **W** matrix. In this case, although the loss of a normal synergy or addition of an abnormal synergy indicated abnormalities of the motor system, the resulting unmatched muscle synergies of the affected limb or the reference limb were often disregarded and not factored into the computation of the similarity index. On the other hand, in prior studies that relied on unidirectional fitting for evaluation, either a set of reference synergies (**W**_R_) was fit into a post-stroke EMG (**D**_S_) or a set of post-stroke synergies (**W**_S_) was fit into a reference EMG (**D**_R_), with a higher fitting R^2^ indicating more normalcy. In this case, a change in dimensionality after stroke may paradoxically result in an increase in fitting R^2^. For instance, if stroke results in smaller dimensionality because of synergy merging, fitting **W**_R_ into **D**_S_ would likely result in a high R^2^ because **W**_R_ spans a larger data subspace that explains noise signals better while perfectly explaining the variances of the merged synergies. Hence, a greater abnormality in muscle synergies may result in a higher similarity index value.

The issue above can be solved by fitting muscle synergies into EMG in both directions (i.e., fitting **W**_R_ into **D**_S_ *and* fitting **W**_S_ into **D**_R_), and computing an index that takes into account the R^2^ resulting from both directions. Therefore, we computed BFRR_W_ to evaluate the normalcy of the **W** matrix:

$${BFRR}_{W}=avg\left[f\left(\frac{{R^{2}}_{W_{R}\to D_{S}}}{{R^{2}}_{W_{S}\to D_{S}}}\right),f\left(\frac{{R^{2}}_{W_{S}\to D_{R}}}{{R^{2}}_{W_{R}\to D_{R}}}\right)\right],\;f\left(x\right)=\left\{\begin{array}{c} x,\;\;x\leq 1\\ 1,\;\;x>1 \end{array}\right.$$.

We note that some studies [17,18,19,21,22,23,28] addressed the dimensionality problem above by assuming that the stroke-affected and reference limbs had the same number of muscle synergies. But this assumption may not hold for all stroke survivors, especially those with severe motor impairment [15].

*Index BFRR_C_.*

To evaluate the normalcy of the **C** matrix, some prior studies compared the activation profiles of the stroke-affected and reference limbs using methods such as Pearson correlation [19] or cross-correlation [21,22], with prior matching of the corresponding muscle synergies using the maximum scalar product method. However, the maximum scalar product method may fail to match a post-stroke synergy to the correct reference synergy because a post-stroke synergy does not necessarily resemble its pre-stroke form (and thus would not result in a high scalar product), especially after synergy merging or fractionation. It is therefore possible that the subsequent analysis compared the activation profiles of incorrectly matched synergies. Other studies used a different approach, such as fitting a set of reference synergies [25] or a set of shared synergies between the affected and reference limbs [26] into the EMGs of the affected and reference limbs, and comparing the resulting activation profiles using methods like cross-correlation [25] or Wilcoxon rank-sum test [26]. However, the activation profiles resulting from fitting a different set of muscle synergies may not fully reflect the real activation status of muscle synergies post-stroke.

Here, we reason that bidirectional fitting of the **C** matrix (i.e., fitting **C**_R_ into **D**_S_ *and* fitting **C**_S_ into **D**_R_) can more accurately assess the normalcy of the **C** matrix than previous approaches. Similar to BFRR_W_, we computed BFRR_C_ using bidirectional fitting of the **C** matrix:

$${BFRR}_{C}=avg\left[f\left(\frac{{R^{2}}_{C_{R}\to D_{S}}}{{R^{2}}_{C_{S}\to D_{S}}}\right),f\left(\frac{{R^{2}}_{C_{S}\to D_{R}}}{{R^{2}}_{C_{R}\to D_{R}}}\right)\right],\;f\left(x\right)=\left\{\begin{array}{c} x,\;\;x\leq 1\\ 1,\;\;x>1 \end{array}\right.$$.

${BFRR}_{C}$ does not require prior matching of muscle synergies (because it treats the **C** matrix as a component derived from the EMG that is separate from the **W** matrix) and takes into account the entire activation profiles of the limbs under comparison. Also, ${BFRR}_{C}$ directly compares the original **C** matrices of the stroke-affected and reference limbs without assuming the same **W** matrix for both limbs, thus more accurately reflecting the post-stroke abnormalities in the neural commands.

*Indices MI*, *FI.*

Computationally, merging refers to the existence of a linear combination of reference synergies which explains a post-stroke synergy better than any single reference synergy [15] (Supplementary Figure S1B). On the contrary, fractionation refers to the existence of a linear combination of post-stroke synergies which explains a reference synergy better than any single post-stroke synergy [15] (Supplementary Figure S1C). Computationally, merging refers to the existence of a linear combination of reference synergies which explains a post-stroke synergy better than any single reference synergy [15]. On the contrary, fractionation refers to the existence of a linear combination of post-stroke synergies which explains a reference synergy better than any single post-stroke synergy [15]. We used MI and FI to quantify the extent of merging and fractionation by calculating the percentage increase in similarity (measured by scalar product, SP) between a set of post-stroke synergies and the cluster centroids of healthy subjects’ synergies when merging and fractionation were considered, respectively:

$$MI\;or\;FI=\frac{\sum {SP}_{recon}-\sum {SP}_{ref}}{\sum {SP}_{ref}}=\frac{\sum {SP}_{recon}}{\sum {SP}_{ref}}-1$$.

*Indices ITV_BFRR_W_*, *ITV_BFRR_C_.*

Previous research indicated that the inter-task variability of the muscle coordination patterns is greater in stroke survivors with better motor performance than those with worse performance [45]. However, it remains unclear whether such inter-task variability is attributable to the **W** or **C** matrix. To investigate the origin of the variability of muscle patterns across movement tasks, for each stroke survivor, we calculated the inter-task variability (ITV) of task-specific **W** and **C** by calculating BFRR_W_ and BFRR_C_ between every pairwise combination of the 8 tasks performed by the same stroke-affected limb, respectively. The averages across the $C_{2}^{8}=28$ values of BFRR_W_ and BFRR_C_ between task comparisons were taken as our index ITV_BFRR_W_ and ITV_BFRR_C_. Since BFRR is a measure of similarity instead of variability, a higher value of ITV_BFRR_W_ or ITV_BFRR_C_ indicates smaller variability in **W** or **C** between tasks.

*Indices DO*, *MEA.*

Since we observed more oscillations in the activation profiles of more severely impaired stroke survivors, we computed an index DO to quantify the degree of oscillation. Specifically, we used two additional matrices **C**_max_ and **C**_min_ to delineate the upper and lower borders of the oscillation in the C matrix, and computed DO by dividing the area resulting from the oscillatory modulation by the total area under the curve of **C**:

$$DO=\frac{\sum {(C}_{max}-C_{min})}{\sum C}$$.

Moreover, we computed index MEA to evaluate the average magnitude of the synergies’ activation that is independent of the oscillatory component:

$$MEA=\frac{\sum C_{min}}{n*t},$$

where *n* is the number of muscle synergies and *t* is the number of time points.

*Indices BFRR_C_ (mod)*, *ITV_BFRR_C_* (*mod*).

We further calculated two additional indices, BFRR_C_ (mod) and ITV_BFRR_C_ (mod), to evaluate the normalcy of the **C** matrix and the inter-task variability in the **C** matrix when the oscillatory component of **C** is disregarded.

### 2.5. Statistical Analysis

All data analysis was performed in Matlab (R2023a). We used the Lilliefors test to evaluate if a sample followed a normal distribution. For paired samples, we assessed group differences by the paired *t*-test (for normal samples) or Wilcoxon signed-rank test (for non-normal samples). For independent samples, we assessed group differences by the independent *t*-test or one-way ANOVA (for normal samples), or the Mann–Whitney U test or Kruskal–Wallis (KW) test (for non-normal samples). If the result of the ANOVA or KW test was significant, we performed a post hoc Tukey–Kramer multiple comparison to determine the significance of each group combination. We set the level of significance as *α* = 0.05.

Pearson correlation was used to assess the linear relationship between two variables. Multiple linear regression was used to determine how much of the variance of the dependent variable could be accounted for by multiple independent variables. Stepwise multiple linear regression (p_enter_ = 0.05, p_remove_ = 0.10) was used to select the appropriate input variables that contribute to the prediction of the output variable.

## 3. Results

### 3.1. All Muscle Synergy Indices (MSIs) Correlated with Motor Impairment Post-Stroke

We began our investigation by visually comparing the muscle synergies and activation profiles of severely impaired stroke survivors (FMA(A) ≤ 10) with those of healthy subjects (Figure 2) to derive insights on how objective MSIs may be best formulated. For instance, when examining the muscle synergies of subjects S11 (FMA(A) = 6/36) and H12 (healthy), we observed that S11 had a greater number of muscle synergies, which were also more fragmented (i.e., sparser in representation) (Figure 2A). In addition, the activation profiles of S11 appeared more oscillatory and also more irregularly shaped in time, and had a smaller activation magnitude, especially when the oscillation area was disregarded (Figure 2B). In another pair of stroke survivor (S25, FMA(A) = 9/36) and healthy subject (H1), we observed that S25 had fewer varieties of synergy selections deployed across tasks since the patient employed the exact same synergy combination (i.e., synergies 1 + 4 + 5 + 7) in both task 3 and task 7, and almost the same combination (i.e., synergies 2 + 3 + 4 + 5 + 6 ± 7) in tasks 4, 5, and 6 (Figure 2C). In contrast, H1 employed a wide variety of synergy combinations that overlap to a much smaller extent across tasks. The above synergy differences are described in more detail in Supplementary Note S3.

**Figure 3:**
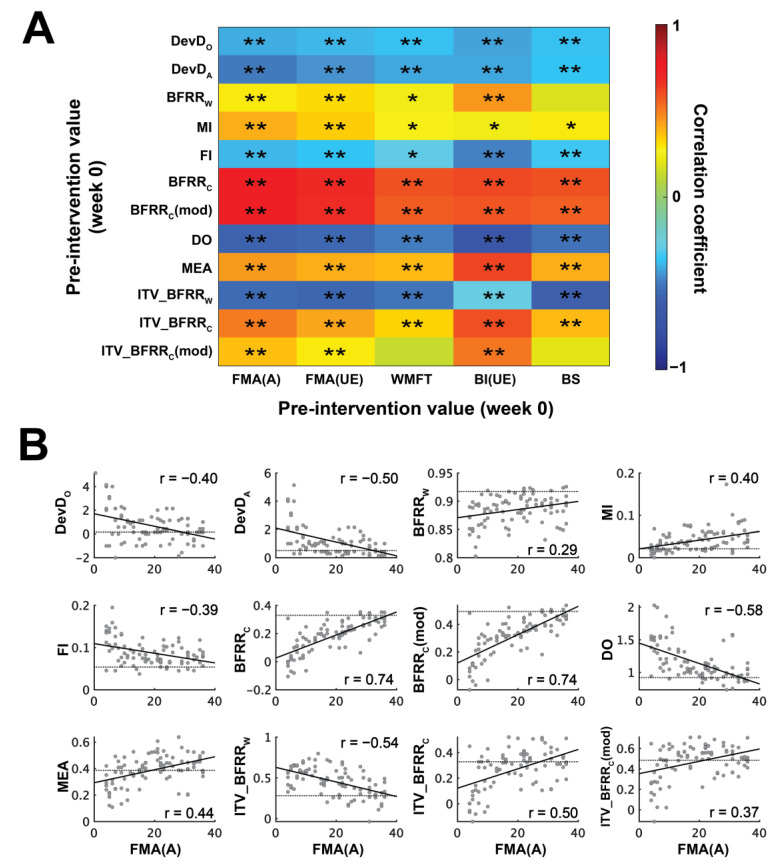
Muscle synergy indices (MSIs) are impairment-relevant. (**A**) Pearson correlation between 12 MSIs and 5 clinical scores at week 0. *: *p* < 0.05; ** *p* < 0.01. (**B**) Scatterplots of MSIs and FMA(A). The dashed line in each scatterplot represents the normative value for each MSI (i.e., the median of healthy subjects).

Motivated by the above observations, we formulated 12 MSIs (Table 1) to quantify the muscle synergy features that differed between stroke survivors and healthy subjects and computed them for all subjects in both groups. For all MSIs except the merging index (MI), values from the severely impaired stroke survivors (FMA(A) ≤ 10) were significantly different from their respective normative values from healthy subjects (*p* < 0.05). The Pearson correlation between the MSIs and the clinical scores were also significant (*p* < 0.05) in 57 out of 60 (from 12 MSIs × 5 scores) instances (Figure 3A). These results suggest that our MSIs were able to capture aspects of post-stroke muscle synergy changes that correlated with the severity of motor impairment and functional disability as indicated by the clinical scores.

As examples, we show in Figure 3B scatterplots of the 12 MSIs against the FMA(A) of all stroke survivors. We note that for all MSIs except MI, the difference between the post-stroke and normative values of MSIs was the greatest at low clinical scores, but for MI, the difference was minimal at low clinical scores and maximal at high clinical scores, which explains why the MI was the only MSI whose values of severely impaired stroke survivors did not differ from those of healthy subjects but still correlated with clinical scores.

### 3.2. Effects of Acupuncture on Muscle Synergy Restoration

To investigate the effect of acupuncture on post-stroke motor recovery, we first compared the absolute changes and realized recovery of clinical scores between acupuncture (Acu) and control interventions (ShamAcu or NoAcu). We calculated the realized recovery of a clinical score by dividing the change in score by the maximum possible score improvement. There was no significant group difference in the absolute change in clinical scores (Table 2) or realized recovery at both weeks 2 and 4 (*p* > 0.10) except for score BS. For this score’s realized recovery, at week 2, the Acu group achieved significantly more recovery (median = 0.14) than the ShamAcu (median = 0) and NoAcu (median = 0) groups (KW test: Chi-sq(2) = 6.83, *p* = 0.033).

**Figure 4:**
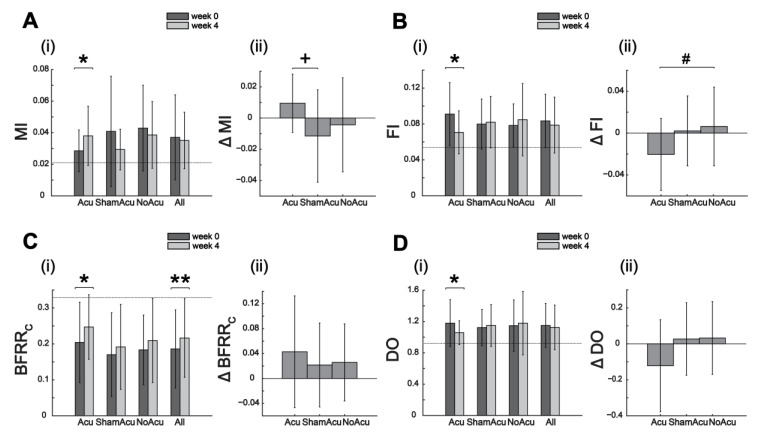
Acu group (n = 21) experienced more changes in muscle synergy indices (MSIs) than the ShamAcu (n = 21) and NoAcu (n = 17) groups, particularly in (**A**) MI, (**B**) FI, (**C**) BFRR_C_, and (**D**) DO. For each MSI, we (**i**) compared the pre-intervention (i.e., week 0) and post-intervention (i.e., week 4) values of each group by paired *t*-test or Wilcoxon signed-rank test (*: *p* < 0.05; **: *p* < 0.01; the dashed line represents the median baseline value for the respective MSI), and (**ii**) compared the 4-week changes of the three interventions by one-way ANOVA or Kruskal–Wallis test (if the result was significant, a post hoc Tukey–Kramer multiple comparison was performed to determine the significance of each group combination). Acupuncture was the only intervention that resulted in significant changes in the four MSIs (*: *p* < 0.05). The changes in MI and FI induced by Acu were significantly different from those by ShamAcu and NoAcu (MI: *p* = 0.040, one-way ANOVA; FI: *p* = 0.043, one-way ANOVA). Multiple comparisons showed that Acu resulted in a greater increase in MI than ShamAcu (^+^: *p* = 0.034) and a greater reduction in FI than NoAcu (^#^: *p* = 0.059).

For MSIs, statistically significant group differences were found for the 4-week changes in MI, FI, and MEA (Figure 4, Table 2). For MI change, the Acu group (mean = 0.01) had a greater increase than the ShamAcu (mean = −0.01) and NoAcu (mean = 0) groups (one-way ANOVA: F(2,56) = 3.40, *p* = 0.040). For FI change, Acu (mean = −0.02) had a greater reduction than ShamAcu (mean = 0) and NoAcu (mean = 0.01) (one-way ANOVA: F(2,56) = 3.34, *p* = 0.043). For MEA change, Acu (mean = 0.04) had a greater increase than ShamAcu (mean = −0.02) and NoAcu (mean = −0.03) (one-way ANOVA: F(2,56) = 2.99, *p* = 0.058).

We then analyzed the longitudinal changes in clinical scores and MSIs induced by each intervention by comparing the pre- and post-intervention values group by group. Intriguingly, while for the five clinical scores, all groups (Acu, ShamAcu, and NoAcu) showed significant improvement at week 4 (15 of 15 instances), for the MSIs, changes were significant in only 7 of 36 comparisons (Table 2). Specifically, Acu induced significant changes in five MSIs (DevD_o_, MI, FI, BFRR_C_, DO), whereas ShamAcu and NoAcu, in zero and two MSIs (ITV_BFRR_C_, ITV_BFRR_C_ (mod)), respectively (Table 2).

**Figure 5:**
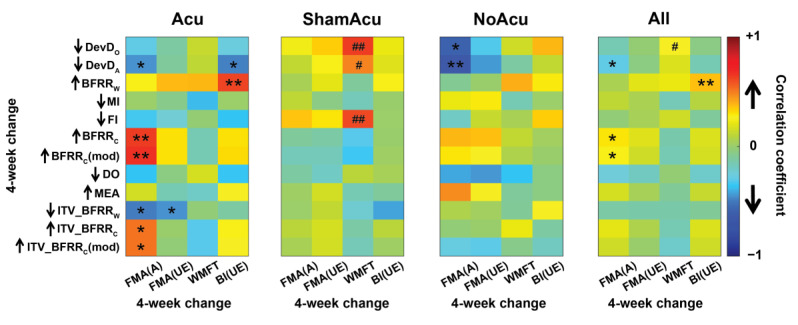
Pearson correlation between the 4-week change in muscle synergy indices (MSIs) and the 4-week change in clinical scores for the Acu group (n = 21), ShamAcu group (n = 21), NoAcu group (n = 17), and all RCT participants (n = 59). The direction of recovery of each synergy feature is indicated by an up-arrow or a down-arrow (e.g., an up-arrow means the average normative value from healthy subjects is greater than the average value of stroke survivors). While in many instances, the increase in clinical scores was associated with MSIs changing towards the normative (*: *p* < 0.05; **: *p* < 0.01), in some instances, the increase in clinical scores was associated with MSIs further deviating from the normative (^#^: *p* < 0.05; ^##^: *p* < 0.01).

The above analysis considers the clinical scores and MSIs separately. We next wondered whether the changes in the clinical scores induced by the interventions may themselves correlate with the changes in any MSIs across the subjects of each group. When we examined the correlations between the 4-week change in MSIs and the 4-week change in clinical scores, we found that only 19 of 192 correlations were significant (Figure 5), indicating that improvement in clinical scores was often not accompanied by the restoration of MSIs. Of the 19 significant correlations, half of them (9/19) belonged to the Acu group, with the pre-to-post direction of MSI changes for all 9 instances moving towards the normative as the clinical scores improved. In contrast, only one fourth came from the ShamAcu or NoAcu group (3/19 and 2/19 instances, respectively). For the three instances in ShamAcu that involved DevD_O_, DevD_A_, and FI changes, the increase in clinical scores induced by ShamAcu was associated with these MSIs further deviating from their normative levels.

We hasten to note that the general lack of correlation between the 4-week changes in MSIs and clinical scores, as shown in Figure 5, does not inherently contradict the pre-intervention MSI-clinical score correlations we showed earlier in Figure 3. First, 4-week score changes induced by the interventions occupied considerably smaller ranges (e.g., from −1 to +28 for ΔFMA(UE)) than pre-intervention values (e.g., from 4 to 63 for FMA(UE)), making it less likely to show correlations between changes in MSIs and clinical scores than those between their pre-intervention values. Second, the lack of correlation in 4-week changes may stem from a considerable proportion of stroke survivors attaining clinical improvement by utilizing alternative motor strategies, resulting in increased clinical scores but unimproved muscle synergy indices (Table 2).

Overall, the effects of Acu could be better differentiated from those of ShamAcu and NoAcu with MSI changes or correlations between MSI and clinical score changes than with clinical score changes alone. Improvement in clinical scores after Acu was accompanied by restorations of more MSIs than ShamAcu and NoAcu.

### 3.3. Subjects Assigned by MSI Predictive Models Had Greater Recovery in Gross Motor Control

**Figure 6:**
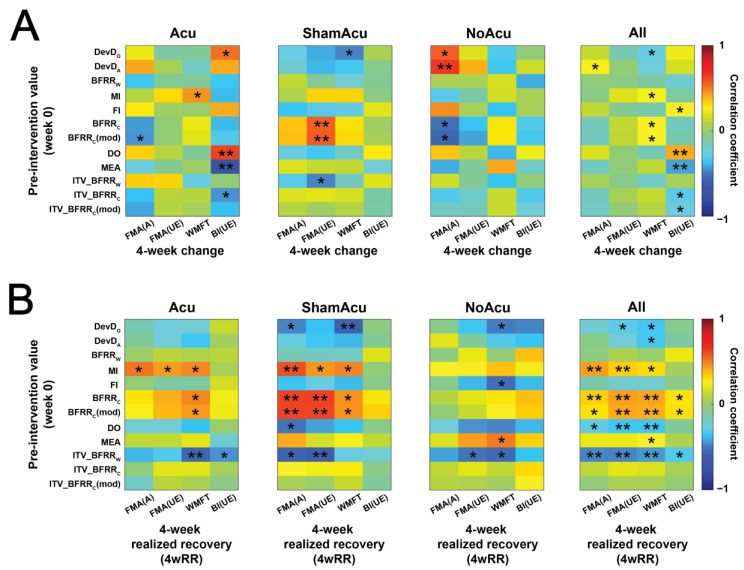
Predictive values of individual muscle synergy indices. (**A**) Pearson correlation between muscle synergy indices (MSIs) at week 0 and the 4-week change in clinical scores for the Acu group (n = 21), ShamAcu group (n = 21), NoAcu group (n = 17), and all RCT participants (n = 59). (**B**) Pearson correlation between MSIs at week 0 and the 4-week realized recovery of clinical scores for the same four groups. *: *p* < 0.05; **: *p* < 0.01.

To investigate whether the pre-intervention MSIs have the potential to predict rehabilitation outcomes, we first computed the Pearson correlation between each MSI at week 0 and the absolute change in each clinical score (Figure 6A) or its realized recovery (Figure 6B) at week 4. MSIs were better at predicting the realized recovery, as a greater proportion of correlations were significant for the realized recovery (48 of 192 instances) than for the absolute score change (24 of 192).

**Figure 7:**
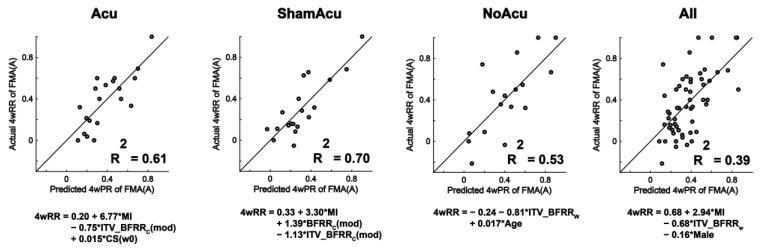
MSI predictive models for FMA(A). MSIs and clinical parameters were selected by stepwise multiple linear regression to predict the 4-week realized recovery of FMA(A) of the Acu group (n = 20), ShamAcu group (n = 19), NoAcu group (n = 17), and all RCT participants (n = 56). A small number of stroke survivors (1 for Acu, 2 for ShamAcu) were excluded because they had full FMA(A) score (i.e., FMA(A) = 36/36) before intervention. CS(w0) refers to the FMA(A) at week 0.

The above results prompted us to perform a stepwise multiple linear regression to systematically select variables from the MSIs, clinical scores, and other parameters (age, gender, affected side, and post-stroke duration) as pre-intervention inputs to predict the realized recovery of clinical scores after intervention. To illustrate, Figure 7 shows the MSI predictive models for FMA(A). The in-sample R^2^ was relatively low when we constructed overall predictive models for all intervention groups (median R^2^ = 0.34) but became higher when we constructed separate predictive models for each intervention group (median R^2^ = 0.51), the smaller sample sizes notwithstanding. Moreover, R^2^ values were considerably smaller when MSIs were not included in the predictive models (R^2^ = 0.26 ± 0.21 with MSIs excluded v.s. R^2^ = 0.41 ± 0.21 with MSIs included).

**Figure 8:**
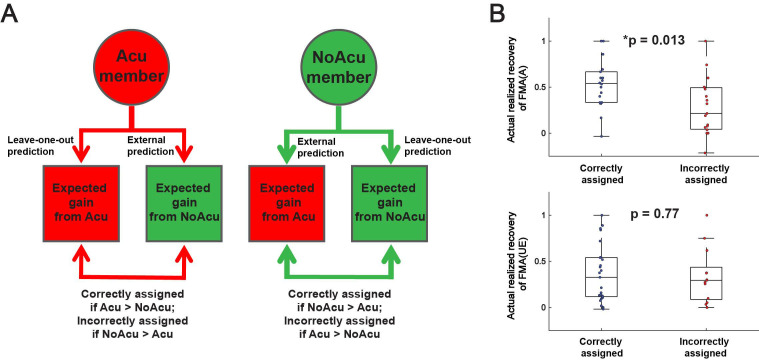
(**A**) For every stroke survivor who received acupuncture (an ‘Acu member’) or no acupuncture (a ‘NoAcu member’), we calculated the realized recoveries of FMA(A) or FMA(UE) expected for acupuncture and no acupuncture to determine if the patient was correctly assigned to the optimal intervention in the RCT. After that, the actual realized recoveries of FMA(A) or FMA(UE) were compared between the correctly assigned and the incorrectly assigned stroke survivors. (**B**) The mean or median actual realized recovery was higher in the correctly assigned group than in the incorrectly assigned group for both FMA(A) and FMA(UE), but the difference was significant only for FMA(A) (independent *t*-test, t(35) = 2.50, *p* = 0.013) but not for FMA(UE) (Mann–Whitney U test, z = 0.29, *p* = 0.77). *: *p* < 0.05.

We evaluated the applicability of the MSI predictive models in determining whether a stroke survivor is suitable for acupuncture by performing a retrospective simulation of the patient stratification process on stroke survivors who received Acu or NoAcu (Figure 8A). First, for every member of both groups, we calculated both the expected realized recovery after acupuncture and no acupuncture, respectively. Specifically, for every Acu member, we calculated the expected realized recovery after acupuncture using the predictive model derived from stepwise multiple linear regression of the data of all other Acu members (i.e., analogous to leave-one-out cross-validation), and that after no acupuncture, using the predictive model developed from the data of the NoAcu group (i.e., analogous to external validation). We calculated the expected realized recovery after acupuncture and no acupuncture for every NoAcu subject analogously. Second, we determined the optimal intervention (i.e., acupuncture or no acupuncture) for each Acu and NoAcu member by choosing the one with a greater expected realized recovery. Third, we classified each Acu and NoAcu member as being correctly or incorrectly assigned to his or her intervention group, as follows. A stroke survivor was deemed correctly assigned by the randomization process in the clinical trial if they indeed received the desired intervention determined by the MSI predictive models, and incorrectly assigned if otherwise. Finally, we determined the validity of the classification based on our predictive models by comparing the actual realized recovery of the correctly and incorrectly assigned subjects.

The above procedure was performed for FMA(A) and FMA(UE). For both scores, the mean or median actual realized recovery was higher in the correctly assigned group than in the incorrectly assigned group, but the difference was significant only for FMA(A) (t(35) = 2.50, *p* = 0.013) but not for FMA(UE) (z = 0.29, *p* = 0.77) (Figure 8B).

## 4. Discussion

Muscle synergy is a compact model representing the coordinative structures utilized by the motor system to construct movement [46]. Previous studies have provided preliminary evidence that abnormalities of muscle synergies correlate with the motor dysfunction resulting from neurological disorders such as stroke [14] (Supplementary Table S1). In this study, we designed and validated 12 MSIs for a wide range of post-stroke synergy characteristics, including the overall normalcy, specific features and task-to-task variability of both the **W** and **C** matrices (Table 1; Figure 2). The fact that the post-stroke values of all our MSIs significantly correlated with multiple clinical scores supports that muscle synergies can effectively reflect the gross impairment status of the motor system. On a related note, the synergy features of our MSIs may be the therapeutic targets of novel therapies such as synergy-based functional electrical stimulation (FES) [47,48,49] or myoelectric interface [29] to restore the pre-stroke motor condition.

Under the framework proposed by Levin et al. [50], motor recovery can happen at the neuronal, performance, and activity levels. While clinical scores assess motor recovery at the activity (e.g., WMFT) or performance (e.g., FMA) level, muscle synergy indices (MSIs) evaluate motor recovery at the neuronal level because muscle synergies correspond to the motor circuits an individual employs to produce movements (Figure 1B). In this light, with consideration of both the clinical scores and muscle synergies, true motor recovery is analogous to an increase in functional scores accompanied by the return of synergy features to their pre-stroke condition, while recovery with compensatory strategies corresponds to motor functional improvement without the MSIs’ return. Although compensation could result in immediate functional gain and serve as a short-term solution, it is less desirable than true recovery in the long run because it can result in maladaptive plasticity and limit the final functional outcome [5,50,51,52]. As a result, motor recovery after stroke ideally should result from the restitution of synergy features rather than the development of compensatory synergy patterns (e.g., the acquisition of new muscle synergies or activating them in a way not observed in nondisabled individuals).

In this study, while all clinical scores in the Acu, ShamAcu, and NoAcu groups improved significantly, only a small subset of MSIs changed towards the normative after intervention (Table 2). Thus, acquiring compensatory strategies (as conceptualized above) presumably plays a non-negligible role in the motor recovery of all three intervention groups, including acupuncture. This result agrees with previous trials in which there was improvement only in the clinical scores but not kinematics [51], and consistent with the fact that basic rehabilitation therapies generally focus more on successful task completion but less on movement quality or the movement’s underlying muscle patterns [52].

However, among the three interventive options, abdominal acupuncture (alongside basic care) was the one the most capable of facilitating the restoration of the MSIs (Figure 4). When we performed Pearson correlation on changes in MSIs with changes in clinical scores, Acu, in contrast to ShamAcu or NoAcu, showed nine correlations with improvement in the clinical scores accompanied by changes in MSIs towards their normative levels (Figure 5). Also, when we considered just MSI changes of individual groups, while ShamAcu and NoAcu only induced significant changes in zero and two MSIs, respectively, Acu induced significant changes in five MSIs (DevD_O_, MI, FI, BFRR_C_, and DO; Table 2). Of the five, four changed towards the normative, while MI deviated further away from the normative (Figure 4). In our multi-group comparisons, Acu was more effective than ShamAcu and NoAcu in promoting the recovery of FI and MEA, but it also induced more merging than the other interventions. Overall, these results indicate that the addition of acupuncture into stroke rehabilitation resulted in the recovery of many dimensions of synergy features (i.e., in terms of dimensionality, fractionation, and synergy activation patterns), although it also permits compensation along specific dimensions, such as merging. Importantly, the above analysis highlights how the MSIs may elucidate the effects of different rehabilitative options in a clinical trial that are not necessarily apparent in clinical score assessments. In our case, the effects of Acu, ShamAcu, and NoAcu would appear similar had the clinical scores been adopted as the only outcome measures.

Should Acu indeed have additional beneficial effects on motor control, why did they not manifest in the clinical assessment? One plausible explanation is that Acu modified muscle synergies in a manner that was only functionally advantageous for those with particular pre-intervention characteristics. This is consistent with the previous observation that acupuncture was only effective if the lesion in the motor pathways was less than half [35]. If so, the therapeutic potential of abdominal acupuncture was likely underestimated by the results of clinical scores in this trial because after randomized subject assignment, the less favorable clinical outcomes of the incorrectly assigned subjects could have obscured the favorable outcomes of the correctly assigned.

In view of this, we developed predictive models to select stroke survivors for acupuncture. Since many pre-treatment MSIs individually correlated with the realized recovery of clinical scores (Figure 6), we tested the feasibility of using multiple MSIs to determine whether each stroke survivor would benefit from acupuncture. Specifically, we used MSIs, clinical data and stepwise multiple linear regression to develop separate predictive models for Acu and NoAcu, respectively, and used them to calculate the expected realized recoveries of both treatment options (i.e., acupuncture or no acupuncture) for each stroke survivor in the Acu and NoAcu groups. As a proof of concept, our retrospective analysis that assigns each subject to the treatment option with the best expected post-treatment outcome demonstrates that our predictive models could lead to a greater realized recovery, especially in gross motor function of the upper limb (Figure 7).

Our results contribute to the prediction for stroke rehabilitation in two potential ways. First, many previous studies have relied on neuroimaging biomarkers to classify stroke survivors into categories with different expected post-rehabilitation potentials [3,4]. Here, we demonstrate that MSIs extracted from EMG-derived muscle synergies could likewise predict post-treatment functional outcomes (Figure 6 and Figure 7). The MSIs may therefore be used as alternative or additional recovery markers that are potentially easier and less expensive to obtain (when compared to other modalities such as structural or functional MRI) once the process of measuring the MSIs is further streamlined [53].

Second, most of the prior studies have focused on predicting the recovery potential of a single rehabilitation option, such as usual care [34,41,54,55,56,57,58] and robotic therapy [59,60]. With this prediction, clinicians may suitably adjust the goals and contents of that particular treatment according to the recovery potential of individual patients. In our study, we explored and compared the predictions of two rehabilitation options, namely, Acu and NoAcu. While past research showed that some rehabilitation options may be similar in nature and therefore the predictive model for one is transferrable to another (e.g., manual ankle stretching vs. robot-assisted stretching [61]), the rehabilitation options in our trial likely had different mechanisms of action and required separate predictive models. As illustrated in Figure 7, the specific predictions made for individual intervention groups (Acu, ShamAcu, and no Acu) were more accurate than the general predictions for stroke survivors of all intervention groups. This likely holds true for many other rehabilitation options, especially the recently developed novel technologies such as transcranial magnetic stimulation (TMS), functional electrical stimulation (FES) and electrical epidural stimulation (EES), which operate on fundamentally distinct theories and principles. Importantly, the workflow we propose in this study to compare different rehabilitation options can potentially be extended to other combinations of treatments beyond Acu versus NoAcu. With more predictive models developed for different treatment modalities, clinicians would be able to identify the best treatment option and individualize strategic plans for the rehabilitation of stroke survivors with diverse pre-rehabilitation characteristics. As these predictive models are implemented in clinical practice, they can be continuously updated and validated using data from new patients. Also, researchers investigating the effectiveness of stroke rehabilitation interventions may use the predictive models to identify suitable clinical trial participants, thereby reducing the variance and achieving equivalent statistical power with fewer subjects.

## 5. Limitations and Future Directions

First, in this study, we used MSI-based predictive models to predict the rehabilitation outcome of specific interventions (i.e., acupuncture vs. no acupuncture) of a specific group of subjects (i.e., stroke survivors at the subacute phase of recovery). Future studies should explore MSI predictions in other interventions or patient groups. Moreover, the predictive power of MSIs was supported here by a retrospective simulation of the patient stratification process. This simulation should be regarded as a preliminary demonstration of the feasibility of an MSI-based stratification, and should certainly be validated by another prospective study, preferably a randomized controlled trial.

Second, the accuracy of our muscle synergy analysis could have been enhanced by incorporating an age-matched and larger-sized control group. Previous studies showed that while the muscle synergies from different age groups are broadly similar, there exist subtle but significant differences between the synergies of older and younger subjects [62,63]. It is possible that age difference may have contributed to the synergy differences between the patient and control groups observed here. However, we think the effect of age is limited in our study given that none of the 12 MSIs significantly correlated with age in either the patient group or the control group. Also, with comparable FMA(UE) scores, the group of stroke survivors aged ≤ 40 (n = 9) and the group aged ≥ 70 (n = 8) were not significantly different in their MSIs.

The sample size of our control group is indeed relatively small when compared with that of the patient group, thus potentially limiting the statistical power and increasing the likelihood of sampling bias. Despite the limited sample size, our data showed significant differences between the severely impaired stroke survivors (FMA(A) ≤ 10) and healthy controls in 11 of the 12 MSIs. This may be attributed to the fact that the sample size of stroke survivors is relatively large (n = 88), and that the synergy features of our control group were relatively homogeneous (the MSIs’ standard deviation of the control group is, on average, 39% of that of the patient group). Indeed, the MSIs considered here may be naturally rather homogenous in healthy subjects, which may reduce the sample size required to achieve positive results. Most importantly, the MSIs of our stroke survivors significantly correlated with the clinical scores in 57 of 60 instances, indicating a relative robustness of our MSIs for motor assessment despite their reliance on a relatively small number of healthy controls as the template for MSIs’ computation.

Third, since we recorded EMGs from the upper limb muscles for gross motor function, it is not unexpected that the MSIs were more closely related to FMA(A) than FMA(UE), with the former reflecting shoulder, arm and forearm functions only while the latter reflecting the functions of the entire upper limb. Indeed, the absolute values of the correlation coefficients of MSIs with FMA(A) at week 0 were significantly greater than those with FMA(UE) (paired *t*-test, t(11) = 2.88, *p* = 0.015). This may also explain why our MSIs were better at predicting the outcome of acupuncture in FMA(A) than FMA(UE). To have a better representation of the entire upper limb, future studies may consider including upper limb muscles for fine motor function, such as those for finger movements.

Last, some of the muscle synergy characteristics of our cohort of stroke survivors differed from those of previous research, possibly because our cohort was mostly in the early subacute phase of stroke, while previous studies mostly recruited chronic patients. In particular, although many previous studies found a decreased number of synergies in severely impaired stroke survivors [15,20,25,26,30], we found an increase in dimensionality instead. In fact, a recent study that analyzed both subacute and chronic stroke survivors also found an increased mean dimensionality [27]. For our patients, merging could be a compensatory strategy for the increased dimensionality, adopted by stroke survivors at an early stage of recovery. This is because while healthy individuals exhibit a minimal degree of merging by definition, a significantly higher level of merging was observed among our stroke survivors (*p* < 0.01), especially those with milder impairments (Figure 2). Since muscle synergies may evolve at different stages through merging and fractionation [15], future studies should consider tracking the muscle synergies of stroke survivors longitudinally from acute to chronic phases of recovery.

## Figures and Tables

**Table 1:** Brief description of the computation of the 12 muscle synergy indices (MSIs) for stroke survivors. A more detailed description can be found in Supplementary Note S2. D_S_ and D_R_: the dimensionality of the stroke-affected and reference limbs; ${R^{2}}_{X\to Y}$: the R^2^ resulting from fitting X into Y (e.g., $W_{R}\to D_{S}$ = fitting the **W** matrix of the reference limb into the EMG of the stroke-affected limb). $\sum {SP}_{ref}$ and $\sum {SP}_{recon}$: the sum of scalar products between matched muscle synergies of the stroke-affected and reference limbs before and after merging or fractionation. $C_{max}$ and $C_{min}$: the matrices that delineate the upper and lower borders of the oscillatory component in the C matrix of the stroke-affected limb. *n* and *t*: number of muscle synergies and EMG time points of the stroke-affected limb. *^a^* Since BFRR is a measure of similarity instead of variability, a higher ITV_BFRR value indicates smaller variability between tasks.

Index	Description
**(1) Dimensionality of the stroke-affected limb**	
*DevD_O_*	Deviation in the Dimensionality from normal (Original value) ${DevD}_{O}=D_{S}-D_{R}$
*DevD_A_*	Deviation in the Dimensionality from normal (Absolute value) ${DevD}_{A}=\left|D_{S}-D_{R}\right|$
**(2) Similarity of the W matrix between the stroke-affected limb and the reference limb**	
*BFRR_W_*	Bidirectional Fitting R^2^ Ratio (BFRR) of the **W** matrix ${BFRR}_{W}=avg\left[f\left(\frac{{R^{2}}_{W_{R}\to D_{S}}}{{R^{2}}_{W_{S}\to D_{S}}}\right),f\left(\frac{{R^{2}}_{W_{S}\to D_{R}}}{{R^{2}}_{W_{R}\to D_{R}}}\right)\right]$, $f\left(x\right)=\left\{\begin{array}{c} x,\;\;x\leq 1\\ 1,\;\;x>1 \end{array}\right.$
**(3) Other features of the W matrix in the stroke-affected limb**	
*MI*	Merging index $MI=\frac{\sum {SP}_{recon}-\sum {SP}_{ref}}{\sum {SP}_{ref}}=\frac{\sum {SP}_{recon}}{\sum {SP}_{ref}}-1$
*FI*	Fractionation index $FI=\frac{\sum {SP}_{recon}}{\sum {SP}_{ref}}-1$
**(4) Similarity of the C matrix between the stroke-affected limb and the reference limb**	
*BFRR_C_*	Bidirectional Fitting R^2^ Ratio (BFRR) of the **C** matrix ${BFRR}_{C}=avg\left[f\left(\frac{{R^{2}}_{C_{R}\to D_{S}}}{{R^{2}}_{C_{S}\to D_{S}}}\right),f\left(\frac{{R^{2}}_{C_{S}\to D_{R}}}{{R^{2}}_{C_{R}\to D_{R}}}\right)\right]$, $f\left(x\right)=\left\{\begin{array}{c} x,\;\;x\leq 1\\ 1,\;\;x>1 \end{array}\right.$
*BFRR_C_(mod)*	Bidirectional Fitting R^2^ Ratio (BFRR) of the modified **C** matrix ${BFRR}_{C}=avg\left[f\left(\frac{{R^{2}}_{{C(mod)}_{R}\to {D(mod)}_{S}}}{{R^{2}}_{{C(mod)}_{S}\to {D(mod)}_{S}}}\right),f\left(\frac{{R^{2}}_{{C(mod)}_{S}\to {D(mod)}_{R}}}{{R^{2}}_{{C(mod)}_{R}\to {D(mod)}_{R}}}\right)\right]$, $f\left(x\right)=\left\{\begin{array}{c} x,\;\;x\leq 1\\ 1,\;\;x>1 \end{array}\right.$
**(5) Other features of the C matrix in the stroke-affected limb**	
*DO*	Degree of Oscillation of the **C** matrix $DO=\frac{\sum {(C}_{max}-C_{min})}{\sum C}$
*MEA*	Magnitude of Effective Activation $MEA=\frac{\sum C_{min}}{n*t}$
**(6) Inter-task variability in the W or C matrix in the stroke-affected limb**	
*ITV_BFRR_W_^a^*	Inter-Task Variability (measured by BFRR) in the **W** matrix ITV_BFRR_W_ is the average across the BFRR_W_ values resulting from the $C_{2}^{8}$ = 28 pairwise comparison of the task-specific muscle synergies of the 8 tasks.
*ITV_BFRR_C_^a^*	Inter-Task Variability (measured by BFRR) in the **C** matrix ITV_BFRR_C_ is the average across the BFRR_C_ values resulting from the $C_{2}^{8}$ = 28 pairwise comparison of the task-specific activation profiles of the 8 tasks.
*ITV_BFRR_C_(mod)^a^*	Inter-Task Variability (measured by BFRR) in the modified **C** matrix ITV_BFRR_C_(mod) is the average across the BFRR_C_(mod) values resulting from the $C_{2}^{8}$ = 28 pairwise comparison of the task-specific modified activation profiles of the 8 tasks.

**Table 2:** Rehabilitation outcomes of the Acu group (n = 21), ShamAcu group (n = 21), NoAcu group (n = 17), and all RCT participants (n = 59). For the clinical scores and muscle synergy indices (MSIs), we compared the pre-intervention (week 0) and post-intervention (week 4) values of each group, and for the clinical scores only, pre-intervention (week 0) and half-completed-intervention (week 2) values, by paired *t*-test or Wilcoxon signed-rank test (†: *p* < 0.08; *: *p* < 0.05; **: *p* < 0.01). The values listed in the table are the average changes across the subjects in each group (mean ± SD). Additionally, we compared the 2- or 4-week changes of the three interventions by one-way ANOVA or Kruskal–Wallis test (^: *p* < 0.06; #: *p* < 0.05).

	Acupuncture	Sham Acupuncture	No Acupuncture	All Groups
	Change in clinical scores (mean ± SD) after 2 weeks			
***FMA(A)***	2.05 ± 2.19 **	2.62 ± 3.05 **	1.41 ± 1.50 **	2.07 ± 2.43 **
***FMA(UE)***	3.95 ± 5.14 **	3.62 ± 4.62 **	2.82 ± 2.28 **	3.51 ± 4.33 **
***WMFT***	5.62 ± 5.07 **	4.71 ± 4.69 **	3.35 ± 2.89 **	4.64 ± 4.49 **
***BI(UE)***	5.43 ± 5.20 **	3.29 ± 5.41 *	2.88 ± 3.61 **	3.93 ± 5.00 **
***BS***	0.86 ± 0.83 **	0.57 ± 0.73 **	0.35 ± 0.84	0.61 ± 0.82 **
	Change in clinical scores (mean ± SD) after 4 weeks			
***FMA(A)***	4.62 ± 4.33 **	5.14 ± 4.80 **	5.71 ± 6.18 **	5.12 ± 5.11 **
***FMA(UE)***	9.76 ± 8.27 **	8.76 ± 7.32 **	9.94 ± 6.68 **	9.46 ± 7.52 **
***WMFT***	12.05 ± 8.40 **	10.33 ± 8.71 **	7.47 ± 5.13 **	10.12 ± 7.94 **
***BI(UE)***	10.86 ± 8.29 **	6.24 ± 7.65 **	8.41 ± 6.80 **	8.51 ± 7.90 **
***BS***	1.52 ± 1.22 **	1.24 ± 1.23 **	1.29 ± 1.45 **	1.36 ± 1.30 **
	Change in muscle synergy indexes (mean ± SD) after 4 weeks			
***DevD_O_***	−0.81 ± 1.56 *	0.19 ± 1.30	0.00 ± 0.97	−0.22 ± 1.39
***DevD_A_***	−0.21 ± 1.30	−0.15 ± 0.90	0.07 ± 0.76	−0.11 ± 1.03
***BFRR_W_***	0.00 ± 0.03	0.00 ± 0.02	−0.01 ± 0.03	0.00 ± 0.03
***MI #***	0.01 ± 0.02 *	−0.01 ± 0.03	0.00 ± 0.03	0.00 ± 0.03
***FI #***	−0.02 ± 0.03 *	0.00 ± 0.03	0.01 ± 0.04	0.00 ± 0.04
***BFRR_C_***	0.04 ± 0.09 *	0.02 ± 0.07	0.03 ± 0.06	0.03 ± 0.07 **
***BFRR_C_ (mod)***	0.05 ± 0.12 †	0.03 ± 0.08	0.03 ± 0.08	0.04 ± 0.10 **
***DO***	−0.12 ± 0.25 *	0.03 ± 0.20	0.03 ± 0.20	−0.02 ± 0.23
***MEA ^***	0.04 ± 0.11	−0.02 ± 0.08	−0.03 ± 0.08	0.00 ± 0.10
***ITV_BFRR_W_***	−0.03 ± 0.16	−0.06 ± 0.13 †	0.00 ± 0.15	−0.03 ± 0.15
***ITV_BFRR_C_***	0.06 ± 0.16	0.00 ± 0.12	0.05 ± 0.10 *	0.04 ± 0.13 *
***ITV_BFRR_C_ (mod)***	0.07 ± 0.19	−0.01 ± 0.14	0.06 ± 0.10 *	0.04 ± 0.16 †

## Data Availability

All data reported in this study as well as the codes developed for the analyses are available to any researcher upon any reasonable request sent to the corresponding authors.

## Supplementary Materials

The following supporting information can be downloaded at: https://www.mdpi.com/article/10.3390/s25103170/s1, Table S1: Muscle synergy indices (for the upper limb) in selected previous studies and our current study; Table S2: Demographic and clinical information of all stroke survivors; Table S3: Demographic and clinical information of individual stroke survivors; Table S4: Task items of clinical scores; Note S1: Inclusion and exclusion criteria for RCT subjects; Note S2: Detailed procedures for MSI computation; Note S3: How muscle synergies changed after stroke; Figure S1: Clustering analysis of the muscle synergies of stroke survivors and healthy subjects. References [15,16,17,18,19,20,21,22,23,24,25,26,27,28,29,30,43,45,64,65] are cited in the supplementary materials.
