# Supplementary material for: Can EMG-Derived Upper Limb Muscle Synergies Serve as Markers for Post-Stroke Motor Assessment and Prediction of Rehabilitation Outcome?
Source: Sensors (Basel). 2025 May 17;25(10):3170. doi: 10.3390/s25103170 (PMC12116162; doi:10.3390/s25103170)
Supplement: Supplementary file 1 [file sensors-25-03170-s001.zip › sensors-3542365-supplementary.pdf]

SUPPLEMENTARY INFORMATION

Supplementary Table S1. Muscle synergy indices (for the upper limb) in selected previous studies and our current study.

	1) Dimensionality	2) Similarity of the W matrix	3) Other features of the W matrix	4) Similarity of the C matrix	5) Other features of the C matrix	6) Inter-task variability in the W or C matrix
**Cheung et al. [16]**<br>**Cheung et al. [15]**	N_{aff}	SP, N_{sh}	MI, FI	/	/	/
**Roh et al. [17]**<br>**Roh et al. [18]**	(universal N_{aff} = 4)<br>(universal N_{aff} = 4)	SP, UDF_W (VAF)	/	/	/	/
**Tropea el al. [19]**	(universal N_{aff} = 4)	SP (dot_{inter})	/	r	/	/
**García-Cossio et al. [20]**	N_{aff}	SP, N_{sh}	MI, FI	/	/	/
**Li et al. [21]**	(universal N_{aff} = 3 for FR and N_{aff} = 4 for LR)	SP (S_V)	/	CC (S_C)	/	/
**Pan et al. [22]**<br>**Pan et al. [23]**	(universal N_{aff} = 3)<br>(universal N_{aff} = 3)	SP	MI	CC	/	/
**Pierella et al. [24]**	N_{aff} (#syn)	SP (dot_{syn})	/	/	/	/
**Irastorza-Landa et al. [25]**	N_{aff} (Num)	SP (P_{index})	MI, FI	CC (FSRI)	/	/
**Park et al. [26]**	N_{aff}	SP (Similarity to 5 mean synergy vectors of control group)	/	Similarity to mean activation coefficients of control group	/	/
**Maistrello et al. [27]**	N_{aff}	SP, N_{sh}	MI (P1)	/	/	/
**Sheng et al. [28] ^**	(universal N_{aff} = 2)	SP, MD	ω	/	TA (T^{PS}),<br>MA (P^{PS})	/
**Seo et al. [29]**	N_{aff}	/	DI	/	/	/
**Funato et al. [30]**	N_{aff}	/	MI (MR)	/	/	/
**Our study**	N_{aff} ($DevD_o$, $DevD_a$)	BDF_W ($BFRR_W$)	MI, FI	BDF ($BFRR_C$, $BFRR_C$ (mod))	DO,<br>MA (MEA)	ITV_W (ITV_BFRR_W),<br>ITV_C (ITV_BFRR_C, ITV_BFRR_C (mod))

The blue color denotes that Pearson or Spearman correlation has been performed between the muscle synergy index and clinical score(s). ^ : Pearson correlation was performed between FMA and a combined score of muscle synergy indices.
Abbreviations: N_{aff} = number of muscle synergies of the affected limb. N_{sh} = number of muscle synergies shared by the affected limb and the reference limb. SP = scalar product (also known as dot product) of normalized vectors (i.e. cosine similarity). MD = Mahalanobis distance. UDF_W = uni-directional fitting of the W matrix. BDF_W = bi-directional fitting of the W matrix. MI = merging index. FI = fractionation index. ω = variation tendency. DI = disparity index. UDF_C = uni-directional fitting of the C matrix. BDF_C = bi-directional fitting of the C matrix. CC = cross-correlation of the C matrix. r = Pearson correlation coefficient. TA = timing of activation. MA = magnitude of activation. DO = degree of oscillation. ITV_W = inter-task variability in the W matrix. ITV_C = inter-task variability in the C matrix.

Supplementary Table S2. Demographic and clinical information of all stroke survivors.

Baseline characteristics (week 0)		Total subjects (n = 88)	RCT subjects (n = 59)	RCT subgroups			P value [a]
				Acu (n = 21)	ShamAcu (n = 21)	NoAcu (n = 17)	
**Demographics (mean ± SD)**	Age (years)	56.23 ± 10.95	57.14 ± 11.14	59.24 ± 8.43	54.76 ± 12.41	57.47 ± 12.47	0.4313
	Gender males/females	57/31	36/23	13/8	13/8	10/7	0.9765
	Post-stroke duration (days)	55.01 ± 34.51	47.59 ± 26.88	39.14 ± 22.06	53.38 ± 30.92	50.88 ± 25.80	0.1935
**Clinical Assessment [b] (mean ± SD)**	FMA(A)	19.15 ± 10.21	19.24 ± 10.01	21.00 ± 9.47	15.95 ± 10.09	21.12 ± 10.09	0.1739
	FMA(UE)	28.10 ± 17.25	26.76 ± 16.46	30.90 ± 17.51	21.57 ± 14.72	28.06 ± 16.39	0.1587
	WMFT	/	26.90 ± 15.52	31.67 ± 15.55	21.00 ± 15.23	28.29 ± 14.26	0.0739
	BI-UE	/	41.47 ± 10.27	40.43 ± 9.19	43.19 ± 9.71	40.65 ± 12.35	0.6406
	BS	/	5.51 ± 2.24	5.76 ± 2.53	5.05 ± 1.91	5.76 ± 2.28	0.5484

[a] P-values resulting from the comparison of the three RCT subgroups (i.e., Acu, ShamAcu, and NoAcu) using one-way ANOVA or KW test. (ANOVA: age, post-stroke duration, FMA(A), WMFT; KW test: gender, FMA(UE), BI-UE, BS). [b] The initial documentation of WMFT, BI(UE), and BS was only available in part of the non-RCT subjects.

Supplementary Table S3. Demographic and clinical information of individual stroke survivors.

	Gender (male = 1)	Age (years)	Type of stroke	Affected side (right = 1)	Post-stroke duration (days)	Site of lesion
**Stroke survivors who received acupuncture (n = 21)**						
**S1**	0	60	Ischemic	1	48	Basal ganglia, centrum semiovale
**S2**	0	45	Ischemic	0	24	Corpus callosum
**S3**	0	66	Ischemic	0	87	Basal ganglia
**S4**	1	72	Ischemic	1	40	Basal ganglia, corona radiata
**S5**	1	56	Ischemic	1	43	Basal ganglia, corona radiata
**S6**	1	64	Ischemic	1	48	Basal ganglia, corona radiata
**S7**	1	48	Ischemic	1	80	Corona radiata
**S8**	1	67	Ischemic	0	29	Basal ganglia, corona radiata
**S9**	1	59	Ischemic	1	28	Basal ganglia
**S10**	1	51	Ischemic	1	30	Frontal lobe, temporal lobe
**S11**	0	58	Ischemic	1	17	Basal ganglia, corona radiata
**S12**	1	61	Ischemic	0	62	Frontal lobe, parietal lobe, temporal lobe
**S13**	0	67	Ischemic	0	14	Basal ganglia, corona radiata
**S14**	1	65	Ischemic	0	16	Frontal lobe, parietal lobe, temporal lobe, occipital lobe
**S15**	0	42	Ischemic	1	40	Basal ganglia, thalamus, cerebral peduncle
**S16**	1	50	Ischemic	1	44	Basal ganglia, corona radiata
**S17**	0	64	Ischemic	0	20	Basal ganglia, corona radiata
**S18**	1	72	Ischemic	0	17	Basal ganglia, corona radiata
**S19**	1	57	Ischemic	0	78	Basal ganglia
**S20**	0	65	Ischemic	1	17	Frontal lobe, parietal lobe, temporal lobe, occipital lobe, insula
**S21**	1	55	Ischemic	0	40	Frontal lobe
**Stroke survivors who received sham acupuncture (n = 21)**						
**S22**	0	55	Ischemic	0	84	Basal ganglia
**S23**	0	70	Ischemic	0	16	Frontal lobe, corona radiata, centrum semiovale
**S24**	0	43	Ischemic	0	16	Basal ganglia
**S25**	1	56	Ischemic	1	68	Basal ganglia
**S26**	1	61	Ischemic	0	150	Frontal lobe, parietal lobe, temporal lobe
**S27**	1	62	Ischemic	0	34	Corona radiata
**S28**	0	31	Ischemic	1	71	Basal ganglia
**S29**	1	56	Ischemic	0	58	Corona radiata
**S30**	0	54	Ischemic	1	27	Basal ganglia, corona radiata
**S31**	1	60	Ischemic	0	42	Basal ganglia
**S32**	0	63	Ischemic	0	16	Cerebral hemisphere
**S33**	1	72	Ischemic	0	25	Centrum semiovale
**S34**	0	58	Ischemic	0	54	Insula, basal ganglia, corona radiata
**S35**	1	46	Ischemic	0	62	Temporal lobe, basal ganglia
**S36**	1	35	Ischemic	1	42	Basal ganglia, corona radiata
**S37**	1	69	Ischemic	0	45	Basal ganglia, internal capsule (posterior limb)
**S38**	1	55	Ischemic	1	79	Basal ganglia
**S39**	1	48	Ischemic	0	74	Basal ganglia, corona radiata
**S40**	1	48	Ischemic	1	43	Thalamus
**S41**	1	75	Ischemic	0	75	Basal ganglia
**S42**	0	33	Ischemic	1	40	Temporal lobe, insula, corpus callosum (genu)
**Stroke survivors who received no acupuncture (n = 17)**						
**S43**	0	69	Ischemic	0	28	Corona radiata
**S44**	1	66	Ischemic	1	69	Frontal lobe, temporal lobe
**S45**	0	57	Ischemic	1	84	Corona radiata, pons
**S46**	1	63	Ischemic	0	81	Basal ganglia
**S47**	1	49	Ischemic	0	34	Basal ganglia
**S48**	0	55	Ischemic	0	15	Basal ganglia, corona radiata
**S49**	1	35	Ischemic	1	14	Basal ganglia
**S50**	1	64	Ischemic	0	83	Corona radiata, thalamus
**S51**	0	75	Ischemic	1	48	Basal ganglia
**S52**	1	63	Ischemic	1	58	Parietal lobe, basal ganglia, corona radiata
**S53**	1	45	Ischemic	0	61	Basal ganglia
**S54**	0	67	Ischemic	1	70	Lateral ventricle
**S55**	1	31	Ischemic	0	30	Basal ganglia, corona radiata
**S56**	1	65	Ischemic	1	14	Basal ganglia
**S57**	0	48	Ischemic	0	82	Frontal lobe, basal ganglia
**S58**	0	71	Ischemic	0	63	Basal ganglia, corona radiata
**S59**	1	54	Ischemic	0	31	Frontal lobe, parietal lobe, temporal lobe, insula, basal ganglia
**Stroke survivors who did not participate in the RCT (n = 29)**						
**S60**	1	62	Ischemic	0	58	Thalamus
**S61**	1	38	Hemorrhagic	1	102	Basal ganglia
**S62**	1	55	Ischemic	1	27	Frontal lobe, basal ganglia, centrum semiovale

**S63**	1	64	Ischemic	0	169	Frontal lobe, parietal lobe
**S64**	1	61	Ischemic	0	168	Frontal lobe, parietal lobe, temporal lobe, insula
**S65**	1	60	Ischemic	0	35	Frontal lobe, basal ganglia, corona radiata
**S66**	1	41	Hemorrhagic	0	68	Basal ganglia
**S67**	0	45	Ischemic	0	24	Corpus callosum (genu)
**S68**	0	43	Ischemic	0	15	Basal ganglia, corona radiata
**S69**	0	46	Hemorrhagic	1	86	Basal ganglia
**S70**	1	62	Ischemic	0	83	Basal ganglia, corona radiata, thalamus
**S71**	1	55	Ischemic	0	82	Basal ganglia, corona radiata
**S72**	1	45	Ischemic	1	29	Basal ganglia, medulla oblongata
**S73**	0	68	Ischemic	1	89	Frontal lobe, parietal lobe, temporal lobe
**S74**	1	68	Ischemic	0	23	Frontal lobe
**S75**	1	57	Ischemic	1	88	Basal ganglia
**S76**	1	60	Ischemic	0	34	Frontal lobe, basal ganglia
**S77**	0	61	Ischemic	0	17	Basal ganglia
**S78**	1	66	Ischemic	0	86	Frontal lobe, parietal lobe, temporal lobe, basal ganglia, corona radiata
**S79**	0	53	Ischemic	0	86	Basal ganglia, pons
**S80**	1	38	Ischemic	1	47	Basal ganglia
**S81**	1	39	Ischemic	1	50	Frontal lobe, parietal lobe, temporal lobe, insula
**S82**	1	36	Hemorrhagic	1	42	Basal ganglia
**S83**	1	44	Hemorrhagic	0	132	Basal ganglia
**S84**	0	60	Ischemic	1	77	Basal ganglia, centrum semiovale
**S85**	0	70	Ischemic	0	14	Frontal lobe (bilateral), basal ganglia, corona radiata (bilateral), central semiovale
**S86**	1	56	Ischemic	1	68	Basal ganglia
**S87**	1	58	Ischemic	1	109	Basal ganglia
**S88**	1	66	Ischemic	0	125	Corona radiata

Supplementary Table S4. Task items of clinical scores.

(A) Fugl-Meyer Assessment of the Upper Extremity (FMA(UE))

Task item	Motion of the task		Score
A. Shoulder/Elbow/Forearm			
1	Reflex activity	Biceps	0 – 2
2		Triceps	0 – 2
3	Flexor synergy	Shoulder retraction	0 – 2
4		Shoulder elevation	0 – 2
5		Shoulder abduction	0 – 2
6		Shoulder external rotation	0 – 2
7		Elbow flexion	0 – 2
8		Forearm supination	0 – 2
9	Extensor synergy	Shoulder adduction/internal rotation	0 – 2
10		Elbow extension	0 – 2
11		Forearm pronation	0 – 2
12	Volitional movement mixing synergies	Hand to lumbar spine	0 – 2
13		Shoulder flexion 0° to 90°	0 – 2
14		Forearm supination/pronation (elbow at 90°, shoulder at 0°)	0 – 2
15	Volitional movement with little or no synergy	Shoulder abduction 0° to 90°	0 – 2
16		Shoulder flexion 90° to 180°	0 – 2
17		Forearm supination/pronation (elbow at 0°, shoulder at 30° to 90°)	0 – 2
18	Normal reflex activity	Biceps, triceps, finger flexors	0 – 2
B. Wrist			
19	Wrist stability (elbow at 90°)		0 – 2
20	Wrist flexion/extension (elbow at 90°)		0 – 2
21	Wrist stability (elbow at 0°)		0 – 2
22	Wrist flexion/extension (elbow at 0°)		0 – 2
23	Wrist circumduction		0 – 2
C. Hand			
24	Finger flexion		0 – 2
25	Finger extension		0 – 2
26	Hook grasp		0 – 2
27	Thumb adduction		0 – 2
28	Pincer grasp		0 – 2
29	Cylinder grasp		0 – 2
30	Spherical grasp		0 – 2
D. Coordination/speed			
31	Tremor		0 – 2
32	Dysmetria		0 – 2
33	Time		0 – 2
Total			0 – 66

Note: FMA(A) consists of task items 1 – 18 (maximum = 36).

(B) Wolf Motor Function Test (WMFT)

Task item	Motion of the task	Score
1	Forearm to table	0 – 5
2	Forearm to box	0 – 5
3	Extend elbow	0 – 5
4	Extend elbow (with weight)	0 – 5
5	Hand to table	0 – 5
6	Hand to box	0 – 5
7	Reach and retrieve	0 – 5
8	Lift can	0 – 5
9	Lift pencil	0 – 5
10	Pick up paper clip	0 – 5
11	Stack checkers	0 – 5
12	Flip cards	0 – 5
13	Turning the key in lock	0 – 5
14	Fold towel	0 – 5
15	Lift basket	0 – 5
Total		0 – 75

(C) Modified Barthel Index (BI)

Task item	Motion of the task	Score
1	Feeding	0 – 10
2	Dressing	0 – 10
3	Grooming	0 – 5
4	Bathing	0 – 5
5	Toileting	0 – 10
6	Chair/bed transfers	0 – 15
7	Bowel control	0 – 10
8	Bladder control	0 – 10
9	Stair climbing	0 – 10
10	Ambulation	0 – 15
10' (if unable to walk)	Ambulation with wheelchair	0 – 5
Total		0 – 100

Note: BI(UE) consists of task items 1 – 6 (maximum = 55).

(D) Brunnstrom Stage (BS)

Stage	Degree of recovery
I	Flaccid paralysis
II	Movements in synergy pattern, emergence of spasticity
III	Voluntary synergy movements, producing movement across joints, increased spasticity
IV	Voluntary movements outside of synergy patterns, decreasing spasticity
V	Developing control of individual or isolated movements
VI	Return to near-normal motor control
	Total: 0 – 12 (6 marks for the upper extremity, and 6 marks for the hand)

Supplementary Note S1. Inclusion and exclusion criteria for RCT subjects

Initial inclusion criteria of the stroke survivors were: (1) First-onset stroke survivors with hemiplegia, with diagnosis of left or right cerebral infarction in the middle cerebral artery supply area confirmed by brain CT or MRI; (2) Aged between 35 and 75 years old; (3) Stroke survivors at 0.5 to 3 months post-stroke presented with stable vital signs; (4) No severe aphasia and cognitive impairment, and were able to understand and execute commands; (5) Able to control sitting balance without external support, with the Brunnstrom stage of hemiplegia (upper limb and hand) being II or above; (6) Agreed to sign the informed consent; (7) The ethics committee agreed to the subject's participation. To enable a greater number of patients to partake in the study, the initial inclusion criteria were modified one year after the study began. Item 1 was amended to include bilateral cerebral infarction, and item 5 was simplified to Brunnstrom stage II or higher (i.e., eliminating the requirement of being able to control sitting balance without external support).

Exclusion criteria were: (1) History of recurrent stroke, subarachnoid hemorrhage, or brain tumor; (2) Contraindication to undergo 3T MR imaging; (3) Claustrophobia; (4) History of severe complications of cardiac, hepatic or renal disease; (5) History of non-compliance with medical interventions; (6) Recent participation in other clinical trials.

Supplementary Note S2. Detailed procedures for MSI computation

1. Deviation in the Dimensionality from normal, Original ($DevD_O$) and Absolute Values ($DevD_A$)

Dimensionality refers to the number of muscle synergies. $DevD_O$ was calculated by subtracting the dimensionality of a healthy subject (D_H) from that of the stroke survivor (D_S), i.e., $DevD_O = D_S - D_H$. $DevD_A$ was calculated by taking the absolute value of the difference in dimensionality, i.e., $DevD_A = |D_S - D_H|$. For each stroke survivor, $DevD_O$ and $DevD_A$ were computed by comparing the dimensionality of the stroke-affected limb with that of the side-matched limb of every healthy subject, and taking the average across the healthy subjects as the final value.

2. Merging Index (MI) and Fractionation Index (FI)

Cheung et al. [15] proposed that merging, fractionation and preservation of the normative muscle synergies are three principal synergy patterns observed in stroke survivors. Computationally, merging happens when there exists a linear combination of more than one healthy synergies that explains a post-stroke synergy better than any single healthy synergy. On the contrary, fractionation occurs when there exists a linear combination of more than one post-stroke synergies that explains a healthy synergy better than any single post-stroke synergy. We used the *k*-means cluster centroids of the healthy muscle synergies (i.e., healthy clusters H1 – H13 explained in the Supplementary Note S3) as a template to quantify the degree of synergy merging and fractionation in stroke survivors by calculating the MI and FI as follows.

To compute MI, we first quantified the similarity between each post-stroke synergy and each healthy cluster centroid by computing the scalar product, after l^2-normalization of the synergy vectors. We then assembled the maximum scalar product value associated with each post-stroke synergy to form a set of reference scalar product values (SP_{ref}) that would be used later for calculating the MI. To identify the merging patterns, we used non-negative least squares (implemented by the Matlab function, lsqnonneg.m) to find the optimal non-negative solution to the problem $Ax \approx b$, where A is the healthy cluster centroids, x is the non-negative linear combination of the healthy clusters, and b is the optimum reconstruction of a post-stroke synergy by merging. This algorithm was repeated to solve for x for every post-stroke synergy. We then calculated the scalar product value between each reconstructed post-stroke synergy (with merging considered) and its corresponding original post-stroke synergy, which resulted in another set of scalar product values (SP_{recon}). Lastly, we computed the MI by

$$\mathrm{MI} = \frac{\sum \mathrm{SP_{recon}} - \sum \mathrm{SP_{ref}}}{\sum \mathrm{SP_{ref}}} = \frac{\sum \mathrm{SP_{recon}}}{\sum \mathrm{SP_{ref}}} - 1,$$

which represents the percentage increase in similarity between the set of post-stroke synergies and the healthy cluster centroids when merging is considered, thus reflecting the degree of merging. For example, when $\Sigma SP_{ref} = 6$ and $\Sigma SP_{recon} = 6.18$, MI = 0.03, indicating that merging results in a 3% increase in scalar product. The minimum value of MI is 0, which indicates that no instance of merging is identified.

The FI was computed in a similar way as MI, but by swapping the relationship between the post-stroke synergies and the healthy cluster centroids. For FI, SP_{ref} includes the maximum scalar product value associated with each healthy cluster centroid. FI was computed analogously by $\mathrm{FI} = \frac{\sum \mathrm{SP_{recon}}}{\sum \mathrm{SP_{ref}}} - 1$.

3. Degree of Oscillation (DO) and Magnitude of Effective Activation of the Activation Profile (MEA)

When we plotted the **C** matrices of stroke survivors, we found that the **C**'s of severe stroke survivors appeared to be more oscillatory than those of healthy subjects (Fig. 2B). Therefore, we developed a way to quantify the degree of oscillatory modulation of the synergies' activation profiles (DO) by using two additional **C** matrices, $\mathbf{C}_{max}$ and $\mathbf{C}_{min}$, to delineate the contour of this component. $\mathbf{C}_{max}$ and $\mathbf{C}_{min}$ were the sliding-window maximum and minimum across time (window width = 5, equivalent to a duration of 100 ms) of **C**, respectively. We set the width of the sliding window to 5 because it could delineate the oscillatory component well. The DO was then calculated by dividing the area resulting from the oscillatory modulation by the total area under the curve of **C**:

$$\mathrm{DO} = \frac{\sum (\mathrm{C_{max}} - \mathrm{C_{min}})}{\sum \mathrm{C}}.$$

To evaluate the average magnitude of the synergies' activation that is independent of the oscillatory component, we calculated the magnitude of "effective" activation (MEA) as follows:

$$\mathrm{MEA} = \frac{\sum \mathrm{C_{min}}}{\mathrm{n} * \mathrm{t}},$$

where n is the number of muscle synergies and t is the number of time points. Note that for the calculations of DO and MEA, all **C** matrices used represent activations of muscle synergies as

unit vectors (i.e., each column of **W** was l^2-normalized and each row of **C** was scaled accordingly).

4. Bidirectional Fitting R^2 Ratio of the **W** or **C** matrix ($BFRR_W$, $BFRR_C$, and $BFRR_C$ (mod))

Previous research examined the similarity between synergy sets from two conditions using the scalar product [64] or unidirectional fitting (i.e., by fitting the synergy set of one condition to the data of the other using NNMF) [65]. The results of either method could be inaccurate if the dimensionalities of the two conditions differ. To illustrate, when scalar product is used to measure post-stroke synergy changes, the post-stroke synergies are first matched to their best-matching healthy synergies, often in a one-to-one manner. Then, scalar product values are computed between each matched synergy pair, and the mean or median scalar product across the pairs is taken to be the final similarity value. If the post-stroke dimensionality is higher than that of the normative, some of the post-stroke synergies would be left unmatched. Although the unmatched synergies may potentially contain information about post-stroke motor impairment, they are not factored into the computation of the final scalar product. On the other hand, when unidirectional fitting is used to measure synergy changes, either a set of healthy synergies ($\mathbf{W}_H$) is fit into a post-stroke EMG ($\mathbf{D}_S$) or a set of post-stroke synergies ($\mathbf{W}_S$) is fit into a healthy EMG ($\mathbf{D}_H$), and the resulting R^2 of fit is used to indicate the similarity of synergy sets. However, any difference in dimensionality between $\mathbf{W}_H$ and $\mathbf{W}_S$ may in some cases result in a paradoxically high R^2 despite substantial synergy differences. For example, if stroke results in a smaller dimensionality because of synergy merging, fitting $\mathbf{W}_H$ into $\mathbf{D}_S$ would likely result in a high R^2 because $\mathbf{W}_H$ spans a larger data subspace that is able to perfectly explain the variances of the merged synergies. The synergy differences attributable to merging are therefore masked by the high R^2.

The above problems can be addressed by cross-fitting **W** in both directions, i.e., fitting $\mathbf{W}_H$ into $\mathbf{D}_S$, and then fitting $\mathbf{W}_S$ into $\mathbf{D}_H$. A bidirectional fit R^2 ratio ($BFRR_W$) can then be calculated as follows:

$$BFRR_W = \mathrm{avg}\left[FRR_{W_H}, FRR_{W_S}\right] = \mathrm{avg}\left[f\left(\frac{R^2 W_{H\to D_S}}{R^2 W_{S\to D_S}}\right), f\left(\frac{R^2 W_{S\to D_H}}{R^2 W_{H\to D_H}}\right)\right],\ f(x) = \begin{cases} x, & x \le 1 \\ 1, & x > 1 \end{cases},$$

where $R^2{}_{W_H\to D_S}$ is the R^2 from fitting $\mathbf{W}_H$ to $\mathbf{D}_S$, $R^2{}_{W_S\to D_S}$ is the R^2 from fitting $\mathbf{W}_S$ to $\mathbf{D}_S$, and similarly, $R^2{}_{W_S\to D_H}$ is the R^2 from fitting $\mathbf{W}_S$ fitting into $\mathbf{D}_H$, and so on. FRR_{W_H} compares ${R_W}^2{}_{H\to S}$ with ${R_W}^2{}_{S\to S}$ to examine the ability of $\mathbf{W_H}$ to explain $\mathbf{D_S}$. If ${R_W}^2{}_{H\to S} > {R_W}^2{}_{S\to S}$, it implies that $\mathbf{W}_H$ can explain noise signals better than $\mathbf{W}_S$ itself since the variance of $\mathbf{D}_S$ unaccounted for by $\mathbf{W}_S$ arises presumably from noise. Therefore, FRR_{W_H} was set to 1 if the (H→S)-to-(S→S) R^2 ratio was >1, but to this ratio if otherwise. FRR_{W_S} was computed analogously. The index $BFRR_W$ was then calculated by taking the average of FRR_{W_H} and FRR_{W_S}. Intuitively, $BFRR_W$ measures the extent to which the sets of muscle synergies of the healthy subject and stroke survivor can explain each other's EMG.

To assess similarity of the synergies' temporal activations, previous studies evaluated the similarity between activation profiles from two conditions by directly comparing them as individual row vectors, usually by cross-correlation. This approach necessitates prior matching of the muscle synergies of both conditions. Some studies, for instance, matched the muscle synergies of both conditions based on the maximum scalar product and then compared the activation profiles of the matched synergies in a pairwise manner. However, the maximum scalar product method may fail to match a post-stroke synergy to the correct healthy synergy because a post-stroke synergy does not necessarily resemble its pre-stroke form, especially after merging or fractionation.

Cross-fitting the **C** matrix in both directions can again help circumvent the matching problem. This is because fitting assesses the potential of the entire **C** matrices to generalize as opposed to the similarity between pairs of individual vectors. Therefore, similar to muscle synergies, we assessed the similarity of activation profiles between a stroke-affected limb and a healthy limb by bidirectional **C** fitting, i.e., fitting the synergies' activation profiles of the healthy limb ($\mathbf{C}_H$) to $\mathbf{D}_S$, and then fitting those of the stroke-affected limb ($\mathbf{C}_S$) to $\mathbf{D}_H$. Bidirectional C fitting requires $\mathbf{C}_H$, $\mathbf{D}_H$, $\mathbf{C}_S$ and $\mathbf{D}_S$ to have the same number of columns. To achieve this, we first considered the $\mathbf{D}_H$ and $\mathbf{D}_S$ episodes recorded from the same task repetition and aligned them in time as follows. We slid the single-episode **D** matrix with a smaller number of columns onto the other single-episode **D** matrix, column by column, from the position where the right half of the smaller **D** overlapped with the larger **D** to the position where only the left half of the smaller overlapped with the larger. Within this range of overlapping, we then identified the position where the two matrices were best aligned with the highest average Spearman correlation, and then isolated the overlapped portions of the two **D** matrices at this position. The two corresponding **C** matrices of the same task repetition were then segmented by the same on- and off-set time points of the **D**s to result in time-aligned **C** and **D** matrices having the same number of columns. This procedure was repeated 40 times (8 tasks x 5 repetitions = 40 episodes). After that, we pooled the time-aligned **C**s and **D**s of all task repetitions and computed $BFRR_C$ by bidirectional **C** fitting:

$$BFRR_C = \mathrm{avg}\left[FRR_{C_H}, FRR_{C_S}\right]$$
$$\mathrm{avg}\left[f\left(\frac{R^2 C_{H\to D_S}}{R^2 C_{S\to D_S}}\right), f\left(\frac{R^2 C_{S\to D_H}}{R^2 C_{H\to D_H}}\right)\right],\ f(x) = \begin{cases} x, & x \le 1 \\ 1, & x > 1 \end{cases},$$

where $R^2{}_{C_H\to D_S}$ is the R^2 from fitting the aligned $\mathbf{C}_H$ to the aligned $\mathbf{D}_S$, and so on. The logic behind this definition is analogous to that behind $BFRR_W$ described above.

The $BFRR_C$ measures the extent to which the synergies' activation profiles of a healthy limb and a stroke-affected limb can explain each other's EMG. As described above, we observed that the activation profiles of many stroke survivors had an oscillatory component (measured by DO) that may lead to a low $BFRR_C$. We wondered whether the other oscillation-independent features of **C** (e.g., the lower-frequency components of the activation and their patterns across muscle synergies and tasks) could also account for the stroke-healthy differences in activation profiles. To remove the effect of **C**

matrix oscillation, we calculated modified $\mathbf{C}_H$ and $\mathbf{C}_S$ as the sliding window mean (window width = 20, equivalent to 400 ms) of the aligned $\mathbf{C}_H$ and $\mathbf{C}_S$, respectively. We set the sliding window width to 20 because it effectively removed oscillations. We then estimated the EMG cleaned of such oscillatory modulations (i.e., modified $\mathbf{D}_H$ and $\mathbf{D}_S$) by multiplying the modified $\mathbf{C}$ matrices by their corresponding $\mathbf{W}$ matrices (i.e., modified $D_i = W_i * \text{modified } C_i,\ i = H$ or S). Lastly, we did bidirectional fitting using the newly modified $\mathbf{C}_H$, $\mathbf{D}_H$, $\mathbf{C}_S$ and $\mathbf{D}_S$, and computed a modified $BFRR_C$ ($BFRR_C$ (mod)). Note that in this scenario both ${R_C}^2_{S\to S}$ and ${R_C}^2_{H\to H}$ equal 1 since both modified D_S and modified D_H were EMGs reconstructed artificially.

For each stroke survivor, all MSIs from bidirectional fitting ($BFRR_W$, $BFRR_C$ and $BFRR_C$ (mod)) were computed by comparing the stroke-affected limb with each of the side-matched healthy limb, and then averaging across the values derived from the healthy subjects as the final value.

5. Inter-Task Variability measured by $BFRR_W$, $BFRR_C$ and $BFRR_C$ (mod) (ITV_ $BFRR_W$, ITV_ $BFRR_C$ and ITV_ $BFRR_C$ (mod))

Lee et al. [45] observed that the inter-task variability of muscle coordination patterns correlated positively with the clinical scores, but did not specify whether such inter-task variability is attributable to the **W** or **C** matrix. To investigate the potential origin of the variability of muscle patterns across movement tasks, for each stroke survivor, we calculated the inter-task variability (ITV) of task-specific **W**, **C**, and the modified **C** by calculating $BFRR_W$, $BFRR_C$, and $BFRR_C$ (mod) between every pairwise combination of the 8 tasks performed by the same stroke-affected limb, respectively. For these three variables, their averages across the $C_2^8 = 28$ values were then taken as an index of inter-task variability for **W** (ITV_$BFRR_W$), **C** (ITV_$BFRR_C$), and **C** (mod) (ITV_$BFRR_C$ (mod)), respectively. The higher the ITV value, the smaller the average distances between the matrices of any two tasks, and the smaller the inter-task variability. Note that this category of MSIs is the only one that uses task-specific muscle synergies instead of overall muscle synergies.

Supplementary Note S3. How muscle synergies changed after stroke

This note describes in detail how different aspects of muscle synergies changed after stroke.

Preservation, merging and fractionation of normative synergies after stroke

The dimensionality (i.e., the number of muscle synergies) of healthy subjects was around eight (7.86 ± 0.53, mean ± SD for both limbs). For the majority of stroke survivors (74 of 88), the dimensionality of the stroke-affected limbs was unchanged or mildly changed, with deviations from normal within 2 ($-2 \leq DevD_O \leq 2$). For the remaining stroke survivors (14 of 88), the dimensionality was greater than normal by more than two ($2 < DevDo \leq 5.14$). Most of these stroke survivors with high dimensionality (12 of 14) were severely impaired (FMA(A) ≤ 10, out of a maximum of 36).

Previous works have identified preservation, merging, and fractionation of the normative muscle synergies as three important patterns of synergies in the stroke-affected upper limb [15], [16]. While for mildly impaired stroke survivors the majority of pre-stroke muscle synergies are expected to be preserved after stroke, in other stroke survivors multiple pre-stroke synergies may merge to become a new synergy with more muscle components. In addition, an individual pre-stroke synergy may also fractionate into multiple new synergies, each with fewer muscle components.

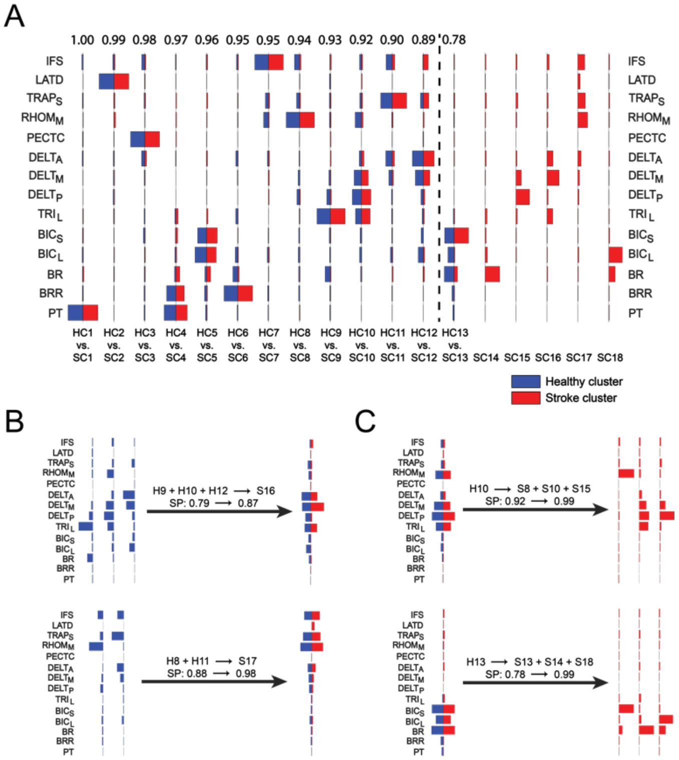

Supplementary Figure S1. Clustering analysis of the muscle synergies of stroke survivors and healthy subjects. (A) The cluster centroids of the muscle synergies of stroke survivors (SC1 – SC18) and those of healthy subjects (HC1 – HC13), matched by maximizing scalar product (SP). The majority of stroke clusters (SC1 – SC12) could be well matched to healthy clusters (HC1 – HC12). (B) Merging of healthy clusters could account for the unmatched stroke clusters S16 and S17. (C) Fractionation of healthy clusters could account for the unmatched stroke clusters SC13, SC14, SC15, and SC18.

To systematically characterize the three post-stroke patterns of muscle synergies, we used *k*-means to group the muscle synergies of stroke survivors and healthy subjects into post-stroke and healthy synergy clusters, respectively, following [15] and [43]. For each number of clusters, *k*-means clustering was conducted 10,000 times. The optimal number of clusters was defined as the minimum number of clusters at which each subject had no more than one muscle synergy represented in the same cluster. As a result, the muscle synergies of stroke survivors and healthy subjects were grouped into 18 post-stroke clusters (SC1 – SC18) and 13 healthy clusters (HC1 – HC13), respectively (Supplementary Fig. S1A). Consistent with previous works, the majority of the stroke survivors' muscle synergies were similar to the normative synergies since 12 of the 18 post-stroke clusters (SC1 – SC12) could be well matched

to a healthy cluster, with scalar product values between cluster centroids ranging from 0.89 to 1.00.

We further investigated whether the remaining post-stroke clusters (SC13 – SC18) could be accounted for by merging or fractionation. Here, for better classification and visualization of these patterns, instances of merging or fractionation were identified only when (1) the coefficients of merging or fractionation were ≥ 0.2, (2) merging or fractionation could lead to a $\geq 5\%$ increase in the scalar-product similarity between the set of healthy clusters and the set of stroke clusters, and (3) the post-merging or fractionation scalar product was ≥ 0.8. Indeed, merging of healthy clusters could account for SC16 and SC17 (Supplementary Fig. S1B) while fractionation of healthy clusters could account for SC13, SC14, SC15 and SC18 (Supplementary Fig. S1C). Similar merging and fractionation patterns were found when comparing the muscle synergies of individual stroke survivors with the healthy clusters.

Merging and fractionation could account for the deviation in dimensionality of the stroke survivors. While the merging index (MI) had a negative correlation with $DevD_O$ ($r = -0.65$, $p < 0.01$), the fractionation index (FI) had a positive correlation ($r = 0.84$, $p < 0.01$). When multiple linear regression was applied, MI and FI together could explain 87% of the variance in $DevD_O$ at week 0 while the change in MI and FI could also explain 87% of the variance in the change in $DevD_O$ after 4 weeks ($p < 0.01$).

Multiple MSIs could quantify post-stroke changes of the synergies' activation profiles

Consistent with our earlier research [16], we found that in our sample of stroke survivors, the synergies' activation profiles were much less preserved after stroke than the muscle synergies themselves. The $BFRR_W$ and $BFRR_C$ indexes assess the overall similarity of **W** and **C** between each stroke survivor and the control subjects, respectively. Although both $BFRR_W$ and $BFRR_C$ of stroke survivors at week 0 were significantly lower than their normative baselines ($p < 0.01$), median $BFRR_C$ of the stroke survivors was reduced by 39% as compared with baseline level, whereas median $BFRR_W$ was reduced by only 3.3%.

As the synergies' activation profiles were altered substantially after stroke, we investigated how such changes could be more precisely characterized as changes of specific features quantifiable by the MSIs. First, index DO, which quantifies the degree of oscillation in **C**, correlated negatively with clinical scores (Fig. 2A, main text). Second, while $BFRR_C$ assesses the overall similarity of the post-stroke **C** to the normative and incorporates the oscillatory component in its assessment, we used another index, $BFRR_C$ (mod), to assess the similarity of the contour of activation without the oscillatory component. We found that both $BFRR_C$ and $BFRR_C$ (mod) correlated positively with the clinical scores. Third, the magnitude of effective activation (MEA) correlated positively with the clinical scores. In addition, the correlation coefficients between MEA and clinical scores (0.45 ± 0.10) were significantly higher than those between the average EMG value (i.e., average of **D**) or the average **C** value and the clinical scores ($p < 0.01$), suggesting that MEA is closely related to post-stroke motor impairment. Overall, the aforementioned findings suggest that the **C**s of stroke survivors differed from those of healthy subjects in that they exhibited more oscillations, more atypical contours, and smaller magnitudes.

Stroke survivors had reduced inter-task variability in the recruited muscle synergies

Lee et al. [45] observed that stroke survivors with greater severity of impairment had smaller inter-task EMG variability. In their study, the EMG of each task was condensed into a root-mean-square (RMS) vector, and the inter-task EMG variability of each stroke survivor was calculated by the squared Euclidean distance between task-specific RMS vectors. Since each task-specific EMG was represented by a single vector, it is unclear whether the reduction in inter-task EMG variability was the result of more rigid task-specific muscle synergies or their activation profiles. Therefore, we extracted task-specific muscle synergies and activation profiles from the EMG of each task, and used ITV_BFRR_W, ITV_BFRR_C and ITV_BFRR_C (mod) to assess the inter-task variability in the **W** and **C** matrices of our stroke survivors. The higher the ITV_BFRR_W, ITV_BFRR_C and ITV_BFRR_C (mod), the lower the inter-task variability in the **W** and **C** matrices.

We found that ITV_BFRR_W correlated negatively with the clinical scores while ITV_BFRR_C and ITV_BFRR_C (mod), positively (Fig. 2A, main text). Therefore, for a more severely impaired stroke survivor, the inter-task variability in synergy recruitment decreased while that in synergy activation increased. Figure 2C (main text) illustrates how the task-specific muscle synergies were more rigid for severely impaired stroke survivors. The upper panel shows the scalar products between the task-specific muscle synergies and overall muscle synergies of a severely impaired stroke survivor (S25, FMA(A) = 9/36). For this subject, synergies 1, 4, 5, and 7 were employed in both task 3 and task 7, while synergies 2 – 6 were employed in tasks 4 – 6. In other words, the subject adopted motor commands within similar subspaces for motor tasks of very different natures. On the other hand, healthy subjects (e.g., H1) utilized different subspaces for different motor tasks (the lower panel).
